# Supplementary material for: Inhibition Underlies Fast Undulatory Locomotion in Caenorhabditis elegans
Source: eNeuro. 2021 Mar 9;8(2):ENEURO.0241-20.2020. doi: 10.1523/ENEURO.0241-20.2020 (PMC7986531; doi:10.1523/ENEURO.0241-20.2020)
Supplement: Extended Data 1 — Code used in this study in three folders: (1) MATLAB program to plot curvature kymograms from hdf5 file generated by Tierpsy. (2) MATLAB program to analyze the change in fluorescence intensity of identifiable body-wall muscle cells or somata of motoneurons. (3) MATLAB code of computational models. Download Extended Data 1, ZIP file. [file enu-eN-NWR-0241-20-s13.zip › 2_CalciumImaging_Code/ImagingAnalyzer02_1_intensity_SOP200807.pdf]

Two-channel Calcium Imaging Analyzer

We used GCaMP, a genetically encoded calcium sensor, alone or with a calcium insensitive red fluorescence protein as a reference for activity imaging in the microfluidic waveform channel.

While calcium recording is made at the motoneuronal soma, the action of the motoneuron is at the neuromuscular junction, along the nematode body. The code calculates the location of the neuromuscular junction of a recorded motoneuron, based on user identification, location of soma, and neuronal morphology (taken from Haspel and O'Donovan 2011); and calculates the phase of the locomotion sysle for that neuromuscular junction based on the dimensions of the sinusoidal microfluidic channel.

Two-channel calcium imaging analysis

After image acquisition with MicroManager, there are three steps for two-channel calcium imaging analysis. The first step is specific to the acquisition software. In the case of MicroManager we needed to change image names to 'GreenImg' and 'RedImg'. The second step is to get the signal from the cell of interest. The third step is to plot traces of calcium signal results.

Step 1. Change the image names.

Usually, we save multiple tiff files under a single folder for each trial. We wrote a Matlab code to change the image name from 'position000' to 'GreenImg' and from 'position001' to 'RedImg' of all the files in each folder. After starting the code, the user needs to select the folder manually, then name changing procedure will start. The name changing codes are listed below:

```
Folder=uigetdir('Select a folder', 'C:\Users\Lan\Desktop\');
cd(Folder);
% The directory of 'Folder' is where folders of images stored
VideoList = ls(Folder);
[m,n] = size(VideoList);

for i = 3:m
    VideoName = VideoList(i,:);
    filepath = strcat(Folder, '\', VideoName);
    cd(filepath);
    inx = 0;
fnameG = ['img_channel000_position000_time' num2str(inx, '%09d') '_z000.tif'];
while exist(fnameG, 'file')

    newnameG =  ['img_' num2str(inx, '%09d') '_GreenImg.tif'];
    fnameR = ['img_channel001_position000_time' num2str(inx, '%09d') '_z000.tif'];
    newnameR =  ['img_' num2str(inx, '%09d') '_RedImg.tif'];
    movefile(fnameG, newnameG);
    movefile(fnameR, newnameR);
    inx=inx+1;
    fnameG = ['img_channel000_position000_time' num2str(inx, '%09d') '_z000.tif'];
end
end
```

Step 2. Track and measure fluorescence signal from calcium sensor and reference protein, calculate ratio and phase (Figure A.5).

1. Open Matlab. Go to the folder '20190405_Tiff Analyzer for CalImg_SynchronizedTwoChannels', run 'ImagingAnalyzer02_1_intensity'.

2. On 'ImagingAnalyzer02intensity' window, change 'fps' (frames per second) if necessary. Select 'ratio: GCaMP/Red' if the calcium sensor is green; select 'ratio: RCaMP/Green' if the calcium sensor is red; select 'dFF: demean' if there is only one channel.

3. Click the 'Load folder of TIFs'. In the open dialog, select the first calcium sensor image of a series of tiffs to be analyzed.

4. Wait for the blue and yellow figure opened on the 'ImagingAnalyer02intensity' window.

5. Change the numbers in 'First frame' and 'Last frame' if necessary.

6. Click the 'Track and plot' button, then a new window with the blue and yellow figure opens.

7. On this new window, use the black crossing cursor to draw a rectangle to circle the cell of interest. If the cell is close to the edge, an error may occur. If so, repeat steps 6 and 7.

8. After drawing a rectangle, a new window appears. On the left bottom of the new window, a magnified view in the selected rectangle appears. In the magnified view at the left bottom, use the black crossing cursor to draw a polygon to circle the cell of interest. Double click to end the polygon drawing.

9. A new magnified view appears with the cell of interest in the next frame. Move the mouse to place the long black crossing cursor at the center of the cell, then click.

10. Then this window renews, and the right bottom magnified view shows the cell of interest in the next magnified frame.

11. Repeat step 9 until the cell of interest goes out of the original view, or click 'q' on the keyboard to stop acquiring fluorescence signal.

12. Go back to 'ImagingAnalyzer02intensity' window, input the name of the cell, click 'Name neuron', choose the head position ('Head left' or 'Head right'). Choose a phase degree ('0 deg', '90 deg', '180 deg', or '270 deg'), then move the long black crossing cursor to the chosen phase degree in the blue and yellow image and click to mark the chosen degree on the image. The name you enter will be used to calculate the location of the neuromuscular junction with respect to the cell body that you tracked, and the location of a known phase will be used to calculate its phase along the locomotor cycle in every frame.

13. Click 'Reset image' to clean the neuron name or chosen degree in the blue and yellow image if needed.

14. Click 'Press for Phase' to change the x-axis of the plot on the left bottom, and to create calcium signal plots and reference protein signal plots over time and undulatory phases.

15. Click 'Save fig and traces' to save plots, screenshot, and data into a mat file.

16. In the mat file, 'NeurName' is the neuron's name. 'UndFreq' is the undulation frequency the cell traveled inside the waveform channel in the microfluidic device. 'Ytrace' is a serial of calcium signal from all the frames analyzed; the value in each frame is the average of the pixels with top 50% intensity in the polygon in step 8 subtracted by the medium intensity of this frame (considered as background signal). 'rYtrace' is a serial of signal from the reference protein channel. 'dFF' is the ratio of calcium signal to reference

protein signal. ‘PhaseSpace’ and ‘Time’ are the corresponding undulation phases and time of these analyzed frames, respectively.

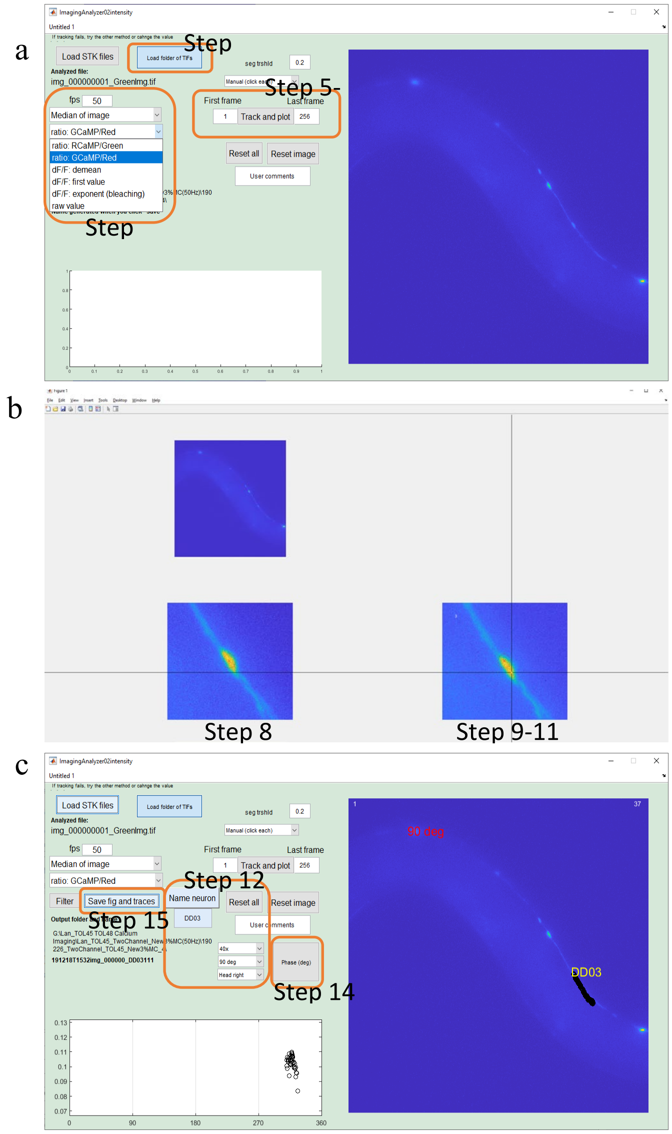

Figure A.5 Graphic user interface (GUI) of ‘Tiff Analyzer for CalImg’, Matlab codes for synchronized two-channel calcium imaging method. (a) On ‘ImagingAnalyzer02intensity’ window, change frame per second (fps); select signal output, ‘ratio:GCaMP/Red’ or ‘ratio:RCaMP/Green’ for two-channel method, or select ‘dF/F:demean’ for single-channel method (Step 2). Click ‘Load folder of TIFs’ to load tiff files (Step 3). Click ‘Track and plot’ to select the cell of interest (Step 5-6). (b) After drawing a rectangle box to circle a region to magnify, draw a polygon to select the cell of interest in the magnified rectangle box at the left bottom (Step 8), then move the long black crossing cursor to the center of the cell in the right bottom box and click (Step 9-11). (c) Input the cell name and click ‘Name neuron’; select a phase and head direction (Step 12). Click ‘Press for Phase’ to create new plots (Step 14). Click ‘Save fig and traces’ to save plots, a screenshot and a mat file (Step 15).

Step 3. Plot calcium imaging in phase space (Figure A.6).

1. Save all the mat files of one type of cell into one folder.

2. Run the Matlab code 'SignalToPhasePlots_Collator' in the folder '20160818_CalImg Analysis'. A window of 'SignalToPhasePlots_Collator' opens.

3. Check 'load folder' and 'Delete the First Frame'. Click 'Load mat files' and select the folder saved with mat files.

4. Edit 'Neur class', 'Direction(F/B)' and 'Genotype'. Change 'Bin size (deg.)' to change the plot binning number, and change 'Error shading' to select the data type for the shaded area.

5. Click 'Plot'. Change the plot type if necessary: 'Scatter', 'Line' or 'Both'.

6. Click 'Save fig and values' to save the screenshot and a mat file.

7. To load another folder of mat files, re-run the Matlab code 'SignalToPhasePlots_Collator' and repeat steps 3-6.

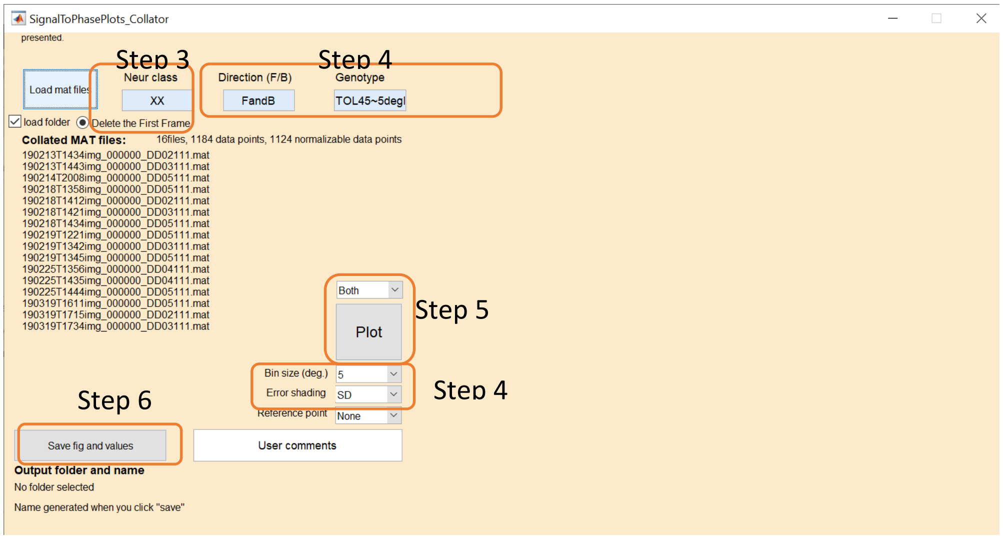

Figure A.6 Operations of Calcium imaging analysis using Matlab code 'SignalToPhasePlots_Collator'. Check 'load folder' and 'Delete the First Frame', and load mat file by clicking 'Load mat files' (Step 3). Edit 'Neur class', 'Direction (F/B)' and 'Genotype'; change 'Bin size (deg.)' and 'Error shading' (Step 4). Click 'Plot' and change plot type (Step 5). Click 'Save fig and values' to save the screenshot and a mat file.
