# Supplementary material for: Inhibition Underlies Fast Undulatory Locomotion in Caenorhabditis elegans
Source: eNeuro. 2021 Mar 9;8(2):ENEURO.0241-20.2020. doi: 10.1523/ENEURO.0241-20.2020 (PMC7986531; doi:10.1523/ENEURO.0241-20.2020)
Supplement: Extended Data 1 — Code used in this study in three folders: (1) MATLAB program to plot curvature kymograms from hdf5 file generated by Tierpsy. (2) MATLAB program to analyze the change in fluorescence intensity of identifiable body-wall muscle cells or somata of motoneurons. (3) MATLAB code of computational models. Download Extended Data 1, ZIP file. [file enu-eN-NWR-0241-20-s13.zip › 2_CalciumImaging_Code/TrackAndMeasure_ImagingAnalyzer/ezyfit/demo/html/efdemo.html]

Discover Ezyfit: A free curve fitting toolbox for Matlab


 


# Discover Ezyfit: A free curve fitting toolbox for Matlab

F. Moisy, 19 nov 2008.

Laboratory FAST, University Paris Sud.

## Contents

- About the Ezyfit Toolbox
- Simple fit: exponential decay
- Initial guesses
- Fitting in linear or in log scale
- Using the fit structure f
- Weigthed fit

## About the Ezyfit Toolbox

The EzyFit toolbox for Matlab enables you to perform simple curve fitting of one-dimensional data using arbitrary fitting
functions. It provides command-line functions and a basic graphical user interface for interactive selection of the data.

## Simple fit: exponential decay

First plot some data, say, an exponential decay

```
plotsample exp nodisp
```

A predefined fit called 'exp' allows you to fit your data:

```
showfit exp
```

```
Equation: y(x) = a*exp(b*x)
     a = 4.3408
     b = -0.22743
     R = 0.99697  (lin)
```

Suppose now you want to use your own variable and function names. Let's fit this data with the function f(t)=a\*exp(-t/tau),
and show the fit with a bold red line:

```
undofit  % deletes the previous fit
showfit('f(t)=a*exp(-t/tau)','fitlinewidth',2,'fitcolor','red');
```

```
Equation: f(t) = a*exp(-t/tau)
     a = 4.3409
     tau = 4.397
     R = 0.99697  (lin)
```

Note that showfit recognizes that t is the variable, and the coefficients of the fit are named a and tau.

If you want to use the values of the coefficients a and tau into Matlab, you need to create these variables into the base
workspace:

```
makevarfit
a
tau
```

```
a =

    4.3409


tau =

    4.3970
```

## Initial guesses

Now suppose you want to fit more complex data, like a distribution showing two peaks. Let's try to fit these peaks with two
gaussians, each of height a, mean m and width s.

```
plotsample hist2 nodisp
showfit('a_1*exp(-(x-x_1)^2/(2*s_1^2)) + a_2*exp(-(x-x_2)^2/(2*s_2^2))');
```

```
Exiting: Maximum number of function evaluations has been exceeded
         - increase MaxFunEvals option.
         Current function value: 369920.454653 

Equation: y(x) = a_1*exp(-(x-x_1)^2/(2*s_1^2))+a_2*exp(-(x-x_2)^2/(2*s_2^2))
     a_1 = 41.052
     a_2 = -2492.7
     s_1 = 1269.4
     s_2 = 13.262
     x_1 = 1062.3
     x_2 = -38.289
     R = 0.32602  (lin)
```

The solver obviously get lost in our 6-dimensional space. Let's help it, by providing initial guesses

```
undofit
showfit('a_1*exp(-(x-m_1)^2/(2*s_1^2)) + a_2*exp(-(x-m_2)^2/(2*s_2^2)); a_1=120; m_1=7; a_2 = 100; m_2=15', 'fitcolor','blue','fitlinewidth',2);
```

```
Equation: y(x) = a_1*exp(-(x-m_1)^2/(2*s_1^2))+a_2*exp(-(x-m_2)^2/(2*s_2^2))
     a_1 = 128.41
     a_2 = 77.126
     m_1 = 6.9929
     m_2 = 14.783
     s_1 = 0.42396
     s_2 = 1.4307
     R = 0.98977  (lin)
```

The result seems to be correct now. Note that only 4 initial guesses are given here; the two other ones, s\_1 and s\_2, are
taken as 1 -- which is close to the expected solution.

## Fitting in linear or in log scale

Suppose you want to fit a power law in logarithmic scale:

```
plotsample power nodisp
showfit power
```

```
Equation: y(x) = a*x^n
     a = 0.4784
     n = 2.494
     R = 0.99934  (log)
```

would you have obtained the same result in linear scale? No:

```
swy    % this shortcut turns the Y-axis to linear scale
showfit('power','fitcolor','red');
```

```
Equation: y(x) = a*x^n
     a = 2.9016
     n = 2.2564
     R = 0.99608  (lin)
```

The value of the coefficients have changed. In the first case, LOG(Y) was fitted, whereas in the second case Y was fitted,
because the Y-axis has been changed.

You may however force showfit to fit LOG(Y) or Y whatever the Y axis, by specifying 'lin' or 'log' in the first input argument:

```
rmfit % this removes all the fits
showfit('power; lin','fitcolor','red');
showfit('power; log','fitcolor','blue');
```

```
Equation: y(x) = a*x^n
     a = 2.9016
     n = 2.2564
     R = 0.99608  (lin)
Equation: y(x) = a*x^n
     a = 0.4784
     n = 2.494
     R = 0.99934  (log)
```

In the equation information, it is specified (lin) or (log) after the R coefficient.

## Using the fit structure f

You can fit your the data without displaying it:

```
x=1:10;
y=[15 14.2 13.6 13.2 12.9 12.7 12.5 12.4 12.4 12.2];
f = ezfit(x,y,'beta(rho) = beta_0 + Delta * exp(-rho * mu);  beta_0 = 12');
```

f is a structure that contains all the informations about the fit:

```
f
```

```
f = 

       name: 'beta(rho)=beta_0+Delta*exp(-rho*mu)'
       yvar: 'beta'
       xvar: 'rho'
    fitmode: 'lin'
         eq: 'beta_0+Delta*exp(-rho*mu)'
          r: 0.9992
      param: {'Delta'  'beta_0'  'mu'}
          m: [3.9949 12.1058 0.3237]
         m0: [1 12 1]
          x: [1 2 3 4 5 6 7 8 9 10]
          y: [1x10 double]
```

From this structure, you can plot the data and the fit:

```
clf
plot(x,y,'r*');
showfit(f)
```

```
Equation: beta(rho) = beta_0+Delta*exp(-rho*mu)
     Delta = 3.9949
     beta_0 = 12.106
     mu = 0.32368
     R = 0.99925  (lin)
```

you can also display the result of the fit

```
dispeqfit(f)
```

```
Equation: beta(rho) = beta_0+Delta*exp(-rho*mu)
     Delta = 3.9949
     beta_0 = 12.106
     mu = 0.32368
     R = 0.99925  (lin)
```

or create the variables in the base workspace

```
makevarfit(f)
beta_0
mu
Delta
```

```
beta_0 =

   12.1058


mu =

    0.3237


Delta =

    3.9949
```

## Weigthed fit

Suppose now we want to fit data with unequal weights, shown here as error bars of different lengths:

```
x =  1:10;
y =  [1.56 1.20 1.10 0.74 0.57 0.55 0.31 0.27 0.28 0.11];
dy = [0.02 0.02 0.20 0.03 0.03 0.10 0.05 0.02 0.10 0.05];
clf, errorbar(x,y,dy,'o');
```

In order to perform a weighted fit on this data, the vectors y and dy have to be merged into a 2-by-N matrix and given as
the second input argument to ezfit. Compare the results for the usual and weighted fits:

```
fw = ezfit(x, [y;dy], 'exp');
showfit(fw,'fitcolor','red');
f = ezfit(x, y, 'exp');
showfit(f,'fitcolor','blue');
```

```
Equation: y(x) = a*exp(b*x)
     a = 2.0017
     b = -0.2519
     R = 0.98832  (lin)
Equation: y(x) = a*exp(b*x)
     a = 2.0071
     b = -0.24013
     R = 0.99067  (lin)
```

The red curve (weighted fit) tends to go through the data with smaller error bars.

Published with MATLAB® 7.6
