# Supplementary material for: Inhibition Underlies Fast Undulatory Locomotion in Caenorhabditis elegans
Source: eNeuro. 2021 Mar 9;8(2):ENEURO.0241-20.2020. doi: 10.1523/ENEURO.0241-20.2020 (PMC7986531; doi:10.1523/ENEURO.0241-20.2020)
Supplement: Extended Data 1 — Code used in this study in three folders: (1) MATLAB program to plot curvature kymograms from hdf5 file generated by Tierpsy. (2) MATLAB program to analyze the change in fluorescence intensity of identifiable body-wall muscle cells or somata of motoneurons. (3) MATLAB code of computational models. Download Extended Data 1, ZIP file. [file enu-eN-NWR-0241-20-s13.zip › 2_CalciumImaging_Code/TrackAndMeasure_ImagingAnalyzer/ezyfit/html/axisc.html]

axisc (Ezyfit Toolbox)


|  |  |
| --- | --- |
| **EzyFit Function Reference** | **<< Prev** | **Next >>** |

axisc  
Centered axis  
  
**Description**
```` ```
axisc centers the axis of the current plot. 
axisc X or axisc Y only centers the horizontal or vertical axe.
```

See Also

```
axis0, gridc. 
 
Published output in the Help browser 
   showdemo axisc
``` ````
  

|  |  |
| --- | --- |
| **Previous: axis0** | **Next: axisl** |

  
2005-2014 EzyFit Toolbox 2.42  
  
