# Supplementary material for: Inhibition Underlies Fast Undulatory Locomotion in Caenorhabditis elegans
Source: eNeuro. 2021 Mar 9;8(2):ENEURO.0241-20.2020. doi: 10.1523/ENEURO.0241-20.2020 (PMC7986531; doi:10.1523/ENEURO.0241-20.2020)
Supplement: Extended Data 1 — Code used in this study in three folders: (1) MATLAB program to plot curvature kymograms from hdf5 file generated by Tierpsy. (2) MATLAB program to analyze the change in fluorescence intensity of identifiable body-wall muscle cells or somata of motoneurons. (3) MATLAB code of computational models. Download Extended Data 1, ZIP file. [file enu-eN-NWR-0241-20-s13.zip › 2_CalciumImaging_Code/TrackAndMeasure_ImagingAnalyzer/ezyfit/html/dispeqfit.html]

dispeqfit (Ezyfit Toolbox)


|  |  |
| --- | --- |
| **EzyFit Function Reference** | **<< Prev** | **Next >>** |

dispeqfit  
Display the equation of a fit.  
  
**Description**
```` ```
dispeqfit(F) displays the equation of the fit F in the command window, 
using the settings defined in fitparam. The fit structure F is 
obtained from ezfit. By default, dispeqfit is automatically called from 
FIT when no output argument is specified.
```

Example

```
   plotsample('power') 
   f = ezfit('alpha/x^n'); 
   dispeqfit(f);
```

See Also

```
fitparam, ezfit, showfit, showeqbox 
 
Published output in the Help browser 
   showdemo dispeqfit
``` ````
  

|  |  |
| --- | --- |
| **Previous: dfig** | **Next: docezyfit** |

  
2005-2014 EzyFit Toolbox 2.42  
  
