# Supplementary material for: Inhibition Underlies Fast Undulatory Locomotion in Caenorhabditis elegans
Source: eNeuro. 2021 Mar 9;8(2):ENEURO.0241-20.2020. doi: 10.1523/ENEURO.0241-20.2020 (PMC7986531; doi:10.1523/ENEURO.0241-20.2020)
Supplement: Extended Data 1 — Code used in this study in three folders: (1) MATLAB program to plot curvature kymograms from hdf5 file generated by Tierpsy. (2) MATLAB program to analyze the change in fluorescence intensity of identifiable body-wall muscle cells or somata of motoneurons. (3) MATLAB code of computational models. Download Extended Data 1, ZIP file. [file enu-eN-NWR-0241-20-s13.zip › 2_CalciumImaging_Code/TrackAndMeasure_ImagingAnalyzer/ezyfit/html/docezyfit.html]

docezyfit (Ezyfit Toolbox)


|  |  |
| --- | --- |
| **EzyFit Function Reference** | **<< Prev** | **Next >>** |

docezyfit  
Documention for the Ezyfit toolbox  
  
**Description**
```` ```
docezyfit displays the start page for the Ezyfit toolbox in the help 
browser. In Matlab 7.3 and before (R2006), typing "doc ezyfit" had the 
same result. However, since Matlab 7.4 (R2007a), the doc function 
changed, and this feature is not available anymore, so you have to 
use docezyfit instead. 
 
docezyfit function_name  displays the documention of function_name 
in the help browser. In Matlab 7.3, this is strictly equivalent to 
DOC function_name.
```

Example

```
  docezyfit showfit 
 
Published output in the Help browser 
   showdemo docezyfit
``` ````
  

|  |  |
| --- | --- |
| **Previous: dispeqfit** | **Next: editcoeff** |

  
2005-2014 EzyFit Toolbox 2.42  
  
