# Supplementary material for: Inhibition Underlies Fast Undulatory Locomotion in Caenorhabditis elegans
Source: eNeuro. 2021 Mar 9;8(2):ENEURO.0241-20.2020. doi: 10.1523/ENEURO.0241-20.2020 (PMC7986531; doi:10.1523/ENEURO.0241-20.2020)
Supplement: Extended Data 1 — Code used in this study in three folders: (1) MATLAB program to plot curvature kymograms from hdf5 file generated by Tierpsy. (2) MATLAB program to analyze the change in fluorescence intensity of identifiable body-wall muscle cells or somata of motoneurons. (3) MATLAB code of computational models. Download Extended Data 1, ZIP file. [file enu-eN-NWR-0241-20-s13.zip › 2_CalciumImaging_Code/TrackAndMeasure_ImagingAnalyzer/ezyfit/html/editcoeff.html]

editcoeff (Ezyfit Toolbox)


|  |  |
| --- | --- |
| **EzyFit Function Reference** | **<< Prev** | **Next >>** |

editcoeff  
Edit the coefficients of a fit  
  
**Description**
```` ```
editcoeff opens the Matlab's Array Editor to edit the coefficients 
of the last fit (which is stored in the variable 'lastfit'). This may 
be useful to copy/paste the fit coefficients into a spreadsheet 
(see the setting 'coeffarray' in fitparam to change the row/column 
display). 
 
editcoeff is also available from the item 'Edit Fit Coefficients' in 
the EzyFit menu, and can be automatically called after each fit (see 
the setting 'editcoeffmode' in fitparam). 
 
editcoeff(F) opens the Matlab's Array Editor to edit the coefficients 
of the fit F. (F is a fit structure as returned by ezfit or showfit). 
 
C = editcoeff(...) returns a 2xN cell array, containing the 
coefficients names and values.
```

Example

```
  plotsample('power'); 
  f = ezfit('power'); 
  editcoeff(f);
```

See Also

```
ezfit, showfit, efmenu, fitparam, makevarfit, OPENVAR. 
 
Published output in the Help browser 
   showdemo editcoeff
``` ````
  

|  |  |
| --- | --- |
| **Previous: docezyfit** | **Next: editfit** |

  
2005-2014 EzyFit Toolbox 2.42  
  
