# Supplementary material for: Inhibition Underlies Fast Undulatory Locomotion in Caenorhabditis elegans
Source: eNeuro. 2021 Mar 9;8(2):ENEURO.0241-20.2020. doi: 10.1523/ENEURO.0241-20.2020 (PMC7986531; doi:10.1523/ENEURO.0241-20.2020)
Supplement: Extended Data 1 — Code used in this study in three folders: (1) MATLAB program to plot curvature kymograms from hdf5 file generated by Tierpsy. (2) MATLAB program to analyze the change in fluorescence intensity of identifiable body-wall muscle cells or somata of motoneurons. (3) MATLAB code of computational models. Download Extended Data 1, ZIP file. [file enu-eN-NWR-0241-20-s13.zip › 2_CalciumImaging_Code/TrackAndMeasure_ImagingAnalyzer/ezyfit/html/efmenu.html]

efmenu (Ezyfit Toolbox)


|  |  |
| --- | --- |
| **EzyFit Function Reference** | **<< Prev** | **Next >>** |

efmenu  
Ezyfit menu  
  
**Description**
```` ```
efmenu adds or refreshes the Ezyfit menu for the current figure and 
for all new figures. 
efmenu OFF removes the menu from all the figures and for all new 
figures. 
 
Careful: when the Ezyfit menu is active, all saved figure files (.FIG) 
include the Ezyfit menu. This generates a problem when those figure 
files are opened in a Matlab system which does not have the Ezyfit 
toolbox installed. In order to remove the Ezyfit menu from the figure 
file, use the function remove_efmenu_fig. 
 
If you want to always have the EzyFit menu in your figures, type 
efmenu INSTALL. This will create or update the 'startup.m' file in the 
user directory of your Matlab installation. In addition, at 
each Matlab restart, this will check the last version of the EzyFit 
toolbox on the web (see checkupdate_ef). 
 
In order to cancel the effect of efmenu INSTALL, you need to edit the 
'startup.m' file and to remove manually the 2 lines of code related to 
the Ezyfit toolbox.
```

See Also

```
plotsample, showfit, ezfit, undofit, rmfit, editfit, 
loadfit, checkupdate_ef, remove_efmenu_fig. 
 
Published output in the Help browser 
   showdemo efmenu
``` ````
  

|  |  |
| --- | --- |
| **Previous: editfit** | **Next: evalfit** |

  
2005-2014 EzyFit Toolbox 2.42  
  
