# Supplementary material for: Inhibition Underlies Fast Undulatory Locomotion in Caenorhabditis elegans
Source: eNeuro. 2021 Mar 9;8(2):ENEURO.0241-20.2020. doi: 10.1523/ENEURO.0241-20.2020 (PMC7986531; doi:10.1523/ENEURO.0241-20.2020)
Supplement: Extended Data 1 — Code used in this study in three folders: (1) MATLAB program to plot curvature kymograms from hdf5 file generated by Tierpsy. (2) MATLAB program to analyze the change in fluorescence intensity of identifiable body-wall muscle cells or somata of motoneurons. (3) MATLAB code of computational models. Download Extended Data 1, ZIP file. [file enu-eN-NWR-0241-20-s13.zip › 2_CalciumImaging_Code/TrackAndMeasure_ImagingAnalyzer/ezyfit/html/ezfft.html]

ezfft (Ezyfit Toolbox)


|  |  |
| --- | --- |
| **EzyFit Function Reference** | **<< Prev** | **Next >>** |

ezfft  
Easy to use Power Spectrum  
  
**Description**
```` ```
ezfft(T,U) plots the power spectrum of the signal U(T) , where T is a 
'time' and U is a real signal (T can be considered as a space 
coordinate as well). If T is a scalar, then it is interpreted as the 
'sampling time' of the signal U.  If T is a vector, then it is 
interpreted as the 'time' itself. In this latter case, T must be 
equally spaced (as obtained by LINSPACE for instance), and it must 
have the same length as U. If T is not specified, then a 'sampling 
time' of unity (1 second for instance) is taken. Windowing 
(appodization) can be applied to reduce border effects (see below). 
 
[W,E] = ezfft(T,U) returns the power spectrum E(W), where E is the 
energy density and W the angular frequency (or pulsation) 'omega' 
(e.g. in rad/s). Note that the frequency (e.g. in Hz) is W/(2*pi). 
If T is considered as a space coordinate (say, X), W is a wavenumber 
(usually noted K = 2*PI/LAMBDA, where LAMBDA is a wavelength). 
 
ezfft(..., 'Property1', 'Property2', ...) specifies the properties: 
 'hann'    applies a Hann appodization window to the data (reduces 
           aliasing). 
 'disp'    displays the spectrum (by default if no output argument) 
 'freq'    the frequency f=2*pi*w is displayed instead of the angular 
           frequency omega (this applies for the display only: the 
           output argument remains the angular frequency omega, not the 
           frequency f). 
 'space'   the time series is considered as a space series. This simply 
           renames the label 'omega' by 'k' (wave number) in the plot, 
           but has no influence on the computation itself. 
 'handle'  returns a handle H instead of [W,E] - it works only if the 
           properties 'disp' is also specified. The handle H is useful 
           to change the line properties (color, thickness) of the 
           plot (see the example below). 
 
The length of the vectors W and E is N/2, where N is the length of U 
(this is because U is assumed to be a real signal.) If N is odd, the 
last point of U and T are ignored. If U is not real, only its real part 
is considered. 
 
    W(1) is always 0.  E(1) is the energy density of the average of U 
      (when plotted in log coordinates, the first point is W(2), E(2)). 
    W(2) is the increment of angular frequency (Delta W), given by 
      2*PI/Tmax. 
    W(end), the highest measurable angular frequency, is PI/DT, where 
      DT is the sampling time (Nyquist theorem). 
 
Parseval Theorem (Energy conservation): 
For every signal U, the 'energy' computed in the time domain and in the 
frequency domain are equal, 
    MEAN(U.^2) == SUM(E)*W(2) 
where W(2) is the increment of angular frequency Delta W. 
Note that, depending on the situation considered, the physical 'energy' 
is usually defined as 0.5*MEAN(U.^2). Energy conservation only applies 
if no appodization of the signal (windowing) is used. Otherwise, some 
energy is lost in  the appodization, so the spectral energy is lower 
than the actual one. The amount of energy lost depends on the signal, 
it may be of order 0.5. 
 
As for FFT, the execution time depends on the length of the signal. 
It is fastest for powers of two.
```

Example

```
  simple display of a power spectrum 
   t = linspace(0,400,2000); 
   u = 0.2 + 0.7*sin(2*pi*t/47) + cos(2*pi*t/11); 
   ezfft(t,u);
```

Example

```
  how to change the color of the plot 
   h = ezfft(t,u,'disp','handle'); 
   set(h,'Color','red');
```

Example

```
  how to use the output of ezfft 
   [w,e] = ezfft(t,u,'hann'); 
   loglog(w,e,'b*');
```

See Also

```
FFT 
 
Published output in the Help browser 
   showdemo ezfft
``` ````
  

|  |  |
| --- | --- |
| **Previous: evalfit** | **Next: ezfit** |

  
2005-2014 EzyFit Toolbox 2.42  
  
