# Supplementary material for: Inhibition Underlies Fast Undulatory Locomotion in Caenorhabditis elegans
Source: eNeuro. 2021 Mar 9;8(2):ENEURO.0241-20.2020. doi: 10.1523/ENEURO.0241-20.2020 (PMC7986531; doi:10.1523/ENEURO.0241-20.2020)
Supplement: Extended Data 1 — Code used in this study in three folders: (1) MATLAB program to plot curvature kymograms from hdf5 file generated by Tierpsy. (2) MATLAB program to analyze the change in fluorescence intensity of identifiable body-wall muscle cells or somata of motoneurons. (3) MATLAB code of computational models. Download Extended Data 1, ZIP file. [file enu-eN-NWR-0241-20-s13.zip › 2_CalciumImaging_Code/TrackAndMeasure_ImagingAnalyzer/ezyfit/html/ezfit.html]

ezfit (Ezyfit Toolbox)


|  |  |
| --- | --- |
| **EzyFit Function Reference** | **<< Prev** | **Next >>** |

ezfit  
Fit data with arbitrary fitting function  
  
**Description**
```` ```
ezfit(FUN) fits the active curve with the function FUN. See below for 
the syntax of FUN. If FUN is not specified, 'linear' is used. 
 
By default, the first curve in the active figure is fitted - see 
fitparam to change this default behavior. To fit another curve, select 
it before calling ezfit.  If some data are selected by the "Data 
Brushing" tool (only for Matlab >= 7.6), only those data are fitted. 
 
ezfit(X,Y,FUN) or ezfit(Y,FUN) fits the data (X,Y) (or Y) using the 
function FUN. X and Y must be vectors of equal length. If X is not 
specified, X=[1, 2, 3...] is assumed. 
 
ezfit(X,Y,FUN), where X is a 1-by-N vector and Y is a 2-by-N matrix, 
also specifies the weights for Y(2,:) (error bars). By default, when 
Y is a 1-by-N vector, all the weights are 1. 
 
Note that ezfit only computes the coefficients, but does not display the 
fit. Use showfit to display the fit. 
 
The function string FUN can be: 
   - the name of a predefined fitting function (see below). 
   - the name of a user-defined fitting function (see editfit). 
   - an equation, in the form 'y(x)=...', where 'x' represents the 
     X-data, and all the other variables are parameters to be fitted 
     ('a', 'x_0', 'tau', ...). Example: 'y(x)=a*sin(b*x)'. If the 
     left-hand-side 'y(x)' is not specified, 'x' is taken for the 
     X-Data. All the parameter names are accepted, except Matlab 
     reserved strings ('sin', 'pi', ...) 
 
The predefined fitting functions are: 
   - linear             y = m * x 
   - affine or poly1    y = a*x + b 
   - poly{n}            y = a0 + a1 * x + ... + an * x^n 
   - power              y = c*x^n 
   - sin                y = a * sin (b * x) 
   - cos                y = a * cos (b * x) 
   - exp                y = a * exp (b * x) 
   - log                y = a * log (b * x) 
   - cngauss            y = exp(-x^2/(2*s^2))/(2*pi*s^2)^(1/2) 
   - cfgauss            y = a*exp(-x^2/(2*s^2)) 
   - ngauss             y = exp(-(x-x0)^2/(2*s^2))/(2*pi*s^2)^(1/2) 
   - gauss              y = a*exp(-(x-x0)^2/(2*s^2)) 
'ngauss' is a 2-parameters normalized Gaussian, and 'gauss' is a 
3-parameters non-normalized (free) Gaussian. 'cngauss' and 'cfgauss' 
are centered normalized and centered free Gaussian, respectively. 
 
ezfit is based on Matlab's built-in FMINSEARCH function (Nelder-Mead 
method), which performs an unconstrained nonlinear minimization of 
the SSR (sum of squared residuals) with respect to the various 
parameters. 
 
The correlation coefficient R is defined as (SSR/(SSE+SSR))^(1/2), where 
   SSR = sum ((y_fit - mean(y)).^2)   % sum of squared residuals 
   SSE = sum ((y_fit - y).^2)         % sum of squared errors 
(see http://mathworld.wolfram.com/CorrelationCoefficient.html) 
 
Nonlinear minimization requires starting guesses (or starting estimates) 
for the fit parameters. By default, all the starting guesses are taken 
as 1, or, when using predefined fits (e.g., exp, gauss, power...), the 
starting guesses are determined depending on the range of the data to 
be fitted. However, in most cases, values closer to the expected 
result should be specified to "help" the convergence. It is sufficient 
to choose values that have the correct sign and correct order of 
magnitude, e.g. 0.01, 1, 100... 
 
The starting guesses for the parameters may be specified in two ways: 
  - directly in the string FUN, after the fit definition: 
       'c0 + a*sin(pi*x/lambda); c0=1; a=0.1; lambda=100' 
       ('!' or '$' may also be used instead of ';'). 
  - by specifying them as an additional input argument for ezfit: 
       ezfit(x,y,'c0 + a*sin(pi*x/lambda)',[0.1 1 100]); 
    Note that in this case the parameters must be ordered alphabetically. 
Note that if both methods are used, only the starting guesses given in 
the string FUN are considered. 
 
By default, Y is fitted in linear mode. If you want to fit LOG(Y) 
instead, you must specify the option 'log' to the string FUN, separated 
by the symbol ';' or '$' or '!' (eg, FUN='a*x^n;log'). This is 
specially useful to fit power laws with equally weighted points in a 
log scale.  If nothing specified, the option 'lin' is used.
```

Example

```
     plotsample('power') 
and compare the results of: 
     ezfit('power;lin') 
     ezfit('power;log') 
 
F = ezfit(...) also returns a structure F having the following fields: 
   - name       name of the fit 
   - eq         equation of the fit 
   - param      cell array of strings: names of the parameters 
   - m          values of the coefficients 
   - m0         initial guess for the coefficients 
   - r          correlation coefficient R (Pearson's correlation) 
   - fitmode    'lin' (y is fitted) or 'log' (log(y) is fitted) mode 
 
This structure F can be further used with showfit, dispeqfit, 
showeqbox, makevarfit and editcoeff. 
 
From F, you can get the values of the fitted parameters. If you want to 
create in the current Matlab workspace the variables associated to 
these parameters, use makevarfit (or set the option 'automakevarfit' 
to 'on' in fitparam).
```

Examples

```
  plotsample damposc 
  f = ezfit('u(t) = c + u_a * sin(2*pi*t/T) * exp(-t/tau); T=5; tau=20'); 
  showfit(f); 
 
  plotsample poly2 
  [x,y] = pickdata; 
  f = ezfit(x, y, 'z(v) = poly3'); 
  editcoeff(f); 
 
  plotsample poly2 
  f = ezfit('beta(z) = poly2'); 
  showfit(f, 'fitcolor', 'red', 'fitlinewidth', 2);
```

See Also

```
showfit, plotsample, dispeqfit, editcoeff, 
FMINSEARCH, makevarfit, fitparam. 
 
Published output in the Help browser 
   showdemo ezfit
``` ````
  

|  |  |
| --- | --- |
| **Previous: ezfft** | **Next: fitparam** |

  
2005-2014 EzyFit Toolbox 2.42  
  
