# Supplementary material for: Inhibition Underlies Fast Undulatory Locomotion in Caenorhabditis elegans
Source: eNeuro. 2021 Mar 9;8(2):ENEURO.0241-20.2020. doi: 10.1523/ENEURO.0241-20.2020 (PMC7986531; doi:10.1523/ENEURO.0241-20.2020)
Supplement: Extended Data 1 — Code used in this study in three folders: (1) MATLAB program to plot curvature kymograms from hdf5 file generated by Tierpsy. (2) MATLAB program to analyze the change in fluorescence intensity of identifiable body-wall muscle cells or somata of motoneurons. (3) MATLAB code of computational models. Download Extended Data 1, ZIP file. [file enu-eN-NWR-0241-20-s13.zip › 2_CalciumImaging_Code/TrackAndMeasure_ImagingAnalyzer/ezyfit/html/ezyfit.html]

EzyFit


|  |  |  |
| --- | --- | --- |
| **Version 2.42 25-Mar-2014**     ---  |  |  | | --- | --- | | **Functions:**  - **By Category** - **In Alphabetical Order** | - **Getting Started** - **Frequently Asked Questions** |  ---  **What's New**  - **Release Notes**    Summarizes new features, bug fixes, upgrade issues, etc. - **Known software problems**  ---  **Documentation Set**  - **Installation**    Installation procedure for the toolbox and the EzyFit menu - **Example Session**    Discover EzyFit - **Settings**    Specify options for fit displays, equation styles, legends... - **EzyFit Uninstallation**    Uninstall the EzyFit toolbox, or only the EzyFit Menu  ---  - **Acknowledgements** - **EzyFit Home Page**  ---  This toolbox is covered by the BSD License.  Copyright (c) 2014, Frédéric Moisy. All rights reserved.    Laboratoire FAST.  University Paris Sud, University Pierre et Marie Curie, CNRS.  91405 Orsay Cedex, France.  moisy@fast.u-psud.fr |
