# Supplementary material for: Inhibition Underlies Fast Undulatory Locomotion in Caenorhabditis elegans
Source: eNeuro. 2021 Mar 9;8(2):ENEURO.0241-20.2020. doi: 10.1523/ENEURO.0241-20.2020 (PMC7986531; doi:10.1523/ENEURO.0241-20.2020)
Supplement: Extended Data 1 — Code used in this study in three folders: (1) MATLAB program to plot curvature kymograms from hdf5 file generated by Tierpsy. (2) MATLAB program to analyze the change in fluorescence intensity of identifiable body-wall muscle cells or somata of motoneurons. (3) MATLAB code of computational models. Download Extended Data 1, ZIP file. [file enu-eN-NWR-0241-20-s13.zip › 2_CalciumImaging_Code/TrackAndMeasure_ImagingAnalyzer/ezyfit/html/ezyfit_faq.html]

EzyFit Frequently Asked Questions


|  |
| --- |
| **EzyFit Frequently Asked Questions** |

# EzyFit Frequently Asked Questions

---

  
If you have a question which is not answered here, or a suggestion on how
you would improve this section, please feel free to send an e-mail to
the author, moisy@fast.u-psud.fr.
  
  

1. Why using EzyFit instead of the standard Matlab's "Basic Fitting" tool?
2. Why using EzyFit instead of MathWorks' "Curve Fitting Toolbox"?
3. I have two curves in my figure, and I want to fit only the second one!
4. Why is there no EzyFit menu in my figure?
5. Can I change the fit color, width, etc.?
6. I want to fit only part of my data!
7. I want the fitted parameters of my data, but whithout drawing the curve!
8. How to change the default color/style of the fits?
9. What are the "initial guesses"?
10. What means "(lin)" or "(log)" in the equation box?
11. Can I extrapolate the fitted curve further than my data range?
12. Where are stored the values of the fitted parameters?
13. Can I save a fit equation?
14. Can I save my own settings configuration?
15. How does EzyFit work?
16. Why do I obtain the error 'Maximum number of function evaluations has been exceeded'?
17. How to uninstall the Ezyfit toolbox?
18. Why can't I open a figure saved with the Ezyfit menu?

  


---

1. **Why using EzyFit instead of the standard Matlab's "Basic Fitting" tool?**  
     
   Well, the "Basic Fitting" looks nice, but it only fits polynomials!
2. **Why using EzyFit instead of MathWorks' "Curve Fitting Toolbox"?**  
     
   MathWorks' "Curve Fitting Toolbox" does much more than EzyFit. But EzyFit is
   simpler (you can make it work in 1 minute), and it is free.
   For most usual problems, EzyFit will surely do the job.
3. **I have two curves in my figure, and I want to fit only the second one!**  
     
   First select the curve you want to fit using the pointer (mouse), and then fit it.
4. **Why is there no EzyFit menu in my figure?**  
     
   Type efmenu to have the EzyFit menu in your
   current and all subsequent figures. You may also type
   > ```
   > efmenu install
   > ```

   to have the EzyFit menu by default in all your figures (this will automatically
   run efmenu at each Matlab restart).
5. **Can I change the fit color, width, etc.?**  
     
   Yes, the fit properties and display settings (color, width,
   equation box...) can be changed by specifying directly some 'PropertyName/PropertyValue' pairs,
   when calling showfit, e.g.
   > ```
   > plotsample power
   > showfit('a*x^n; n=-1','fitcolor','red','fitlinestyle','--');
   > showfit('a*x^n+c_0; n=-1','fitcolor','black','fitlinestyle','-.');
   > legend show
   > ```

   See the Settings page for details.  
   You may also change the default settings, which are coded in the
   M-file fitparam.m (click
   here to open the M-file and follow the
   instructions).
6. **I want to fit only part of my data!**  
     
   If you have Matlab >= 7.6, use the "Data Brushing" tool to select the points
   you want to fit, and then call ezfit or showfit.
7. **I want to get the fitted parameters of my data, but whithout drawing the curve!**  
     
   If your data are X,Y, you just obtain the fitted parameters by
   > ```
   > f = ezfit(x,y,'a*ln(x/c)+k');
   > ```

   See this question to see how to get the fitted values
   from f.
8. **How to change the default color/style of the fits?**  
     
   See the options fp.fitcolor, fp.fitlinestyle and fp.fitlinewidth
   in fitparam.
9. **What are the "initial guesses"?**  
     
   For nonlinear fits, you should provide initial guesses for the
   parameters of your fits to "help" the solver to converge towards
   the "good" values. The solver (the fminsearch function)
   starts from the initial guesses and then varies the coefficients in an
   attempt to find the best fit.  
   For example, suppose you have the data
   > ```
   > x=1:10;
   > y=[15 14.2 13.6 13.2 12.9 12.7 12.5 12.4 12.4 12.2];
   > plot(x,y,'*')
   > ```

   which you want to fit with an exponential decay,
   > ```
   > showfit('cste+a0*exp(-x/L)');
   > ```

   You will obtain:which obvisouly is bad. By default, showfit has used
   cste=1, a0=1 and L=1, which is too far away
   from the 'true' coefficients. To specify the intial guess:
   > ```
   > showfit('cste+a0*exp(-x/L); cste=10');
   > ```

   you now obtain:which is clearly much better. In this example, the initial guesses for a0
   and L, which are omitted, are 1.  
   You may also specify the initial guesses like this:
   > ```
   > showfit('cste+a0*exp(-x/L)',[10 1 1]);
   > ```

   In this case, you should specify ALL the initial guesses, in the alphabetical
   order of the parameter names (here L, a0 and cste because
   L is a capital letter).
10. **What means "(lin)" or "(log)" in the equation box?**  
      
    It indicates whether the data itself, say Y, or its logarithm, LOG(Y),
    has been fitted. By default the mean square procedure is applied to Y if the Y-axis of the current
    figure is linear, and
    it is applied LOG(Y) if the Y-axis is logarithmic. For instance, plot a
    power law in log scales,
    > ```
    > plotsample power
    > ```

    If you try
    > ```
    > showfit('power')
    > ```

    the logarithm of the data is fitted. If you turn the Y-axis in linear scale
    (you may use swy for this) and fit again, you will obtain different values.  
    You may also force ezfit to fit Y or LOG(Y) whatever
    the Y-axis, by specifying the optional argument
    > ```
    > showfit('power;log','fitcolor','blue');
    > showfit('power;lin','fitcolor','red');
    > ```

    See the example:In this example, the fit with option log (blue line) "works better"
    when displayed with a logarithmic Y-axis.
11. **Can I extrapolate the fitted curve further than my data range?**  
      
    Yes, see the option fp.extrapol in fitparam.
12. **Where are stored the values of the fitted parameters?**  
      
    Fit your data using
    > ```
    > f = ezfit('a*x+b');
    > ```

    The structure f contains all the informations about your fit. The
    strings 'a' and 'b' of the parameter names are stored in f.param
    and their values are in f.m. This works also with showfit.  
    You may also directly create in the current workspace a set of variables which
    contain the values of the fitted parameters:
    > ```
    > makevarfit(f);
    > ```

    This will create two variables, 'a' and 'b'. You can also set the option
    'automakevarfit = on' in fitparam: This will
    call makevarfit each time ezfit (or showfit) is executed.
13. **Can I save a fit equation?**  
      
    Yes. First, you may define your favorite fit equation as a user-defined fit,
    by typing
    > ```
    > editfit(3,'myspectrum','E(k) = C*k^(-n);  C=0.1; n=2; log');
    > ```

    This will update the file 'userfit.mat' in the EzyFit directory, so
    your fit equation may be re-used in a future session. You can now fit your
    data using this user-defined fit,
    > ```
    > showfit('myspectrum');
    > ```

    Since a fit equation is a simple string, another way is to create a variable,
    > ```
    > myfit = 'E(k) = C*k^(-n);  C=0.1; n=2; log';
    > ```

    and to save it in a Mat-file,
    > ```
    > save('myfits.mat','myfit');
    > ```

    To fit your data with this fit definition, just type
    > ```
    > showfit(myfit);
    > ```
  
14. **Can I save my own settings configuration?**  
      
    Yes. The default settings (color, width, equation box etc.) are coded
    in the file fitparam.m. So you may create a copy of this file
    with your own settings, and you just have to rename the settings file
    you want to use fitparam.m.
  
15. **How does EzyFit work?**  
      
    The core function of the EzyFit toolbox is ezfit, which
    is based on Matlab's built-in FMINSEARCH function (Nelder-Mead
    method). FMINSEARCH performs an unconstrained nonlinear minimization of
    the SSR (sum of squared residuals) with respect to the various parameters.
    The additional function showfit
    simply calls ezfit with graphical output.
  
16. **Why do I obtain the error 'Maximum number of function evaluations has been exceeded'?**  
      
    This error means that FMINSEARCH could not converge towards a solution in a
    reasonable number of
    iterations. There is no way for the moment to change this number
    of iterations in EzyFit. Try to use initial guesses closer to
    the expected values.
  
17. **How to uninstall the Ezyfit toolbox?**  
      
    See the uninstallation procedure here.
  
18. **Why can't I open a figure saved with the Ezyfit menu?**  
      
    If the Ezyfit toolbox has been installed using efmenu,
    all saved figures (.fig files) and GUIs created using GUIDE have
    the Ezyfit menu included (sometimes several occurence of the Ezyfit menu).
    If you try to open the figure or GUI under a Matlab system without the Ezyfit
    toolbox, the error ??? Error using ==> struct2handle
    Undefined function or variable 'efmenu' is issued.
    Use the function remove\_efmenu\_fig
    (introduced in Version 2.40) in order to remove the menu.
    If you want to uninstall the Ezyfit toolbox, or only the Ezyfit menus,
    see here.

  
  

|  |
| --- |
|  |

  
2005-2014 EzyFit Toolbox  
