# Supplementary material for: Inhibition Underlies Fast Undulatory Locomotion in Caenorhabditis elegans
Source: eNeuro. 2021 Mar 9;8(2):ENEURO.0241-20.2020. doi: 10.1523/ENEURO.0241-20.2020 (PMC7986531; doi:10.1523/ENEURO.0241-20.2020)
Supplement: Extended Data 1 — Code used in this study in three folders: (1) MATLAB program to plot curvature kymograms from hdf5 file generated by Tierpsy. (2) MATLAB program to analyze the change in fluorescence intensity of identifiable body-wall muscle cells or somata of motoneurons. (3) MATLAB code of computational models. Download Extended Data 1, ZIP file. [file enu-eN-NWR-0241-20-s13.zip › 2_CalciumImaging_Code/TrackAndMeasure_ImagingAnalyzer/ezyfit/html/ezyfit_func_alpha.html]

Functions -- Alphabetical List (EzyFit Functions)


|  |
| --- |
| **EzyFit Function Reference** |

# Functions -- Alphabetical List

---

  
about\_ef  
axis0  
axisc  
axisl  
checkupdate\_ef  
dfig  
dispeqfit  
editcoeff  
editfit  
efmenu  
eq2ml  
evalfit  
ezfft  
ezfit  
fitparam  
getslope  
getlineinfo  
gridc  
linx  
liny  
loadfit  
loglogpn  
logx  
logy  
makevarfit  
myginput  
pickdata  
plotsample  
rmfit  
remove\_efmenu\_fig  
semilogypn  
showeqbox  
showfit  
showresidual  
showslope  
sw  
swx  
swy  
undofit  
  
  

|  |
| --- |
|  |

  
2005-2014 EzyFit Toolbox  
