# Supplementary material for: Inhibition Underlies Fast Undulatory Locomotion in Caenorhabditis elegans
Source: eNeuro. 2021 Mar 9;8(2):ENEURO.0241-20.2020. doi: 10.1523/ENEURO.0241-20.2020 (PMC7986531; doi:10.1523/ENEURO.0241-20.2020)
Supplement: Extended Data 1 — Code used in this study in three folders: (1) MATLAB program to plot curvature kymograms from hdf5 file generated by Tierpsy. (2) MATLAB program to analyze the change in fluorescence intensity of identifiable body-wall muscle cells or somata of motoneurons. (3) MATLAB code of computational models. Download Extended Data 1, ZIP file. [file enu-eN-NWR-0241-20-s13.zip › 2_CalciumImaging_Code/TrackAndMeasure_ImagingAnalyzer/ezyfit/html/ezyfit_func_cat.html]

Functions -- By Category (EzyFit Functions)


|  |
| --- |
| **EzyFit Function Reference** |

# Functions -- By Category

---

```` ```
Curve Fitting 
  ezfit          - fit data with arbitrary fitting function 
  showfit        - fit the active curve and display it 
  efmenu         - Ezyfit menu 
  plotsample     - Display a sample plot 
  undofit        - Remove the last fit 
  rmfit          - Remove fits 
  fitparam       - Global settings for the EzyFit toolbox 
   
Curve fitting "by eye" 
  getslope       - Get the slope of the current line 
  showslope      - Draw a line with fixed slope 
 
Curve Fitting Tools 
  editcoeff      - Edit the coefficients of a fit 
  makevarfit     - Create variables from the parameters of a fit 
  evalfit        - Evaluate a fit 
  showresidual   - Show fit residual 
  editfit        - Edit a user-defined fit 
  loadfit        - load the predefined and the user-defined fits 
  dispeqfit      - Displays the fit equation in the command window 
  showeqbox      - Displays the fit equation box in the figure 
 
Miscellaneous 
  remove_efmenu_fig - Remove the Ezyfit menu from figure files
  ezfft          - Power spectrum (FFT)
  pickdata       - Pick data from the active curve 
  getlineinfo    - Get information (length, angle) of a segment
  myginput       - Graphical input from mouse 
  about_ef       - display the "About" information of the EzyFit toolbox
  checkupdate_ef - check for update of the EzyFit toolbox
 
Quick change of the axis scales 
  linx           - Turn the X axis to LIN 
  liny           - Turn the Y axis to LIN 
  logx           - Turn the X axis to LOG 
  logy           - Turn the Y axis to LOG 
  swx            - LIN<->LOG swap of the X axis 
  swy            - LIN<->LOG swap of the Y axis 

Some useful plot tools  
  dfig           - Create docked figure window
  gridc          - Centered cross grid 
  axisc          - Centered axis
  axis0          - Include origin in the axis
  axisl          - Include the nearest power of 10 in the axis of a log plot
  loglogpn       - Log-log scale plot for positive and negative data
  semilogypn     - Semilogarithmic  plot for positive and negative data
``` ````
  

|  |
| --- |
|  |

  
2005-2014 EzyFit Toolbox  
