# Supplementary material for: Inhibition Underlies Fast Undulatory Locomotion in Caenorhabditis elegans
Source: eNeuro. 2021 Mar 9;8(2):ENEURO.0241-20.2020. doi: 10.1523/ENEURO.0241-20.2020 (PMC7986531; doi:10.1523/ENEURO.0241-20.2020)
Supplement: Extended Data 1 — Code used in this study in three folders: (1) MATLAB program to plot curvature kymograms from hdf5 file generated by Tierpsy. (2) MATLAB program to analyze the change in fluorescence intensity of identifiable body-wall muscle cells or somata of motoneurons. (3) MATLAB code of computational models. Download Extended Data 1, ZIP file. [file enu-eN-NWR-0241-20-s13.zip › 2_CalciumImaging_Code/TrackAndMeasure_ImagingAnalyzer/ezyfit/html/ezyfit_gettingstarted.html]

EzyFit Getting Started


|  |
| --- |
| **EzyFit Getting Started** |

# EzyFit Getting Started

---

  

The EzyFit toolbox for Matlab enables you to perform simple curve fitting
of one-dimensional data using arbitrary fitting functions. It provides
command-line functions and a basic graphical user interface for interactive
selection of the data.

  

## Why using EzyFit?

There are three ways to perform curve fitting with Matlab: the first
one is using the 'Basic Fitting Interface' of Matlab, the second one is using
fminsearch with anonymous functions, and the third one
is to pay for the
Curve Fitting Toolbox.
However, for usual curve fitting of 1D data, you may find the first solution
rather limited, the second one a little complicated, and the third one quite
expensive...

The EzyFit Toolbox provides a free, simple and efficient way to
perform quick curve fitting with arbitrary (nonlinear) fitting functions:
You just have to type something like showfit('c+a/x^n') and EzyFit gives
you the values for c, a and n and shows you the
curve! EzyFit also provides a basic graphical user interface
to interactively fit selected parts of your data.

  

## First step: fitting data from the EzyFit menu

First plot some sample data by typing
plotsample
in the command window (or simply click here
to display the sample figure).

In the EzyFit menu of the figure window, select Showfit
and
choose an appropriate fitting function to fit the sample data. You may also click
on the link that appears in the command window to choose the default
fitting function associated to the sample plot. If the menu is not present in
your figure window, type efmenu,
or see the step 4 in the installation procedure.

Choose Undo Fit if you want to remove the fit.

If multiple curves are present in the figure,
first select the one you want to fit, and then choose Showfit.

Two additional functions, getslope and
showslope, return the slope of the selected
line (use the menu
Insert > Line), or drag a line of fixed slope, for a fit "by eye"
of your data.

  

## Second step: fitting data from the command window

Although fitting your data from the EzyFit menu is fast and easy,
using the command-line functions is much more powerful, and allows for
using different properties for each fit (See the Settings
page for details).

The key function of the toolbox is ezfit,
which computes the coefficients that fit the data. The function
showfit simply calls the function
ezfit for the active curve, using the
"Data Brushing" tool of Matlab (available since version 7.6).
Type undofit to remove the last fit.

This example fits some noisy data by a power law c \* x^n and plots the result:

> ```
> plotsample power           % a power law
> showfit c*x^n
> ```

This example fits an histogram with a Gaussian, and displays the coefficients in
the Array Editor:

> ```
> plotsample hist           % an histogram
> f = ezfit('gauss');       % fits with a Gaussian
> f                         % displays the fit structure
> showfit(f);               % displays the fit
> editcoeff(f);             % opens the Array Editor
> ```

### More examples?

Have a look to the Sample session for more examples.

See also the Frequently Asked Questions section and
the Function by category section to
learn more about this toolbox.

  
  

|  |
| --- |
|  |

  
2005-2014 EzyFit Toolbox  
