# Supplementary material for: Inhibition Underlies Fast Undulatory Locomotion in Caenorhabditis elegans
Source: eNeuro. 2021 Mar 9;8(2):ENEURO.0241-20.2020. doi: 10.1523/ENEURO.0241-20.2020 (PMC7986531; doi:10.1523/ENEURO.0241-20.2020)
Supplement: Extended Data 1 — Code used in this study in three folders: (1) MATLAB program to plot curvature kymograms from hdf5 file generated by Tierpsy. (2) MATLAB program to analyze the change in fluorescence intensity of identifiable body-wall muscle cells or somata of motoneurons. (3) MATLAB code of computational models. Download Extended Data 1, ZIP file. [file enu-eN-NWR-0241-20-s13.zip › 2_CalciumImaging_Code/TrackAndMeasure_ImagingAnalyzer/ezyfit/html/ezyfit_install.html]

EzyFit Installation
s


|  |
| --- |
| **EzyFit Installation** |

# EzyFit Installation

---

  

EzyFit needs Matlab 7.0 or higher. It has been
tested under 7.0 to 8.3 (R2014a), but mainly under Windows.
The command-line functions (e.g. ezfit, showfit...)
work equally well on all systems.
However graphical operations (e.g. getslope, showslope...)
may not be fully stable,
especially on non-Windows systems.

1. Download and unzip the EzyFit Toolbox in a directory somewhere in
your system. For instance, in a Windows 7 installation,
the directory Documents/MATLAB/ezyfit may be a
good location. Do **NOT** install
the toolbox in the directory of the Matlab application (Program Files/Matlab directory
in Windows).
If you upgrade from an older version, first empty the previous directory.

2. Select 'Set Path' (available in the menu File in Matlab 7, or in the tab Home in Matlab 8). In the dialog box, click on 'Add Folder' (**NOT** 'with subfolders')
and select the ezyfit directory. Click on 'Save' and 'Close'.

3. If you want to always have the Ezyfit menu in your figures, type
efmenu install. This will create or update your 'startup.m' file in the main user directory
of your Matlab installation.

Note: If you upgrade Matlab and you want to use your previous
Ezyfit installation, you just have to follow the steps 2-3.

See also: Ezyfit Uninstallation

  

|  |
| --- |
|  |

  
2005-2014 EzyFit Toolbox  
