# Supplementary material for: Inhibition Underlies Fast Undulatory Locomotion in Caenorhabditis elegans
Source: eNeuro. 2021 Mar 9;8(2):ENEURO.0241-20.2020. doi: 10.1523/ENEURO.0241-20.2020 (PMC7986531; doi:10.1523/ENEURO.0241-20.2020)
Supplement: Extended Data 1 — Code used in this study in three folders: (1) MATLAB program to plot curvature kymograms from hdf5 file generated by Tierpsy. (2) MATLAB program to analyze the change in fluorescence intensity of identifiable body-wall muscle cells or somata of motoneurons. (3) MATLAB code of computational models. Download Extended Data 1, ZIP file. [file enu-eN-NWR-0241-20-s13.zip › 2_CalciumImaging_Code/TrackAndMeasure_ImagingAnalyzer/ezyfit/html/ezyfit_knownbugs.html]

EzyFit Known software problems


|  |
| --- |
| **EzyFit Known software problems** |

# EzyFit Known software problems

---

  

If you find a bug not documented here, first have a look
to the updated EzyFit
Known software problems page online.
If it is still undocumented, send an e-mail to
moisy@fast.u-psud.fr.

  

- **Figures and GUIs created by GUIDE always have the Ezyfit menu included.**  
  If the Ezyfit toolbox has been installed using efmenu,
  all saved figures (.fig files) and GUIs created using GUIDE have
  the Ezyfit menu included (sometimes several occurence of the Ezyfit menu).
  If you try to open the figure or GUI under a Matlab system without the Ezyfit
  toolbox, the error ??? Error using ==> struct2handle
  Undefined function or variable 'efmenu' is issued.
  Use the function remove\_efmenu\_fig
  (introduced in Version 2.40) in order to remove the menu.
  If you want to uninstall the Ezyfit toolbox, or only the Ezyfit menus,
  see here.
- **New user defined fits created from a given window are not automatically
  updated in the EzyFit menu of other window(s).**  
  Select the other window(s) and chose Refresh EzyFit menu in the menu,
  or type efmenu in the Command Window.
- **When loading a fig file which already contains the EzyFit menu, a
  second EzyFit menu is added.**  
  Type efmenu, this will remove the extra
  menu.
- **Sometimes showslope displays a
  line with a slope slightly different from the required one.**  
  This problem originates from the time
  delay between the mouse click and the pointer location inquiry, especially
  when the mouse is moved quickly. Move slowly.

  
  

|  |
| --- |
|  |

  
2005-2014 EzyFit Toolbox  
