# Supplementary material for: Inhibition Underlies Fast Undulatory Locomotion in Caenorhabditis elegans
Source: eNeuro. 2021 Mar 9;8(2):ENEURO.0241-20.2020. doi: 10.1523/ENEURO.0241-20.2020 (PMC7986531; doi:10.1523/ENEURO.0241-20.2020)
Supplement: Extended Data 1 — Code used in this study in three folders: (1) MATLAB program to plot curvature kymograms from hdf5 file generated by Tierpsy. (2) MATLAB program to analyze the change in fluorescence intensity of identifiable body-wall muscle cells or somata of motoneurons. (3) MATLAB code of computational models. Download Extended Data 1, ZIP file. [file enu-eN-NWR-0241-20-s13.zip › 2_CalciumImaging_Code/TrackAndMeasure_ImagingAnalyzer/ezyfit/html/ezyfit_releasenotes.html]

EzyFit Release Notes


|  |
| --- |
| **EzyFit Release Notes** |

# EzyFit Release Notes

---

  

See also the EzyFit known software problems section.

  

|  |  |  |
| --- | --- | --- |
| V2.42 | 2014/03/25 | **Bug fix**  - efmenu install did not work properly under Windows   **Minor change**:  - Fix some details in the documentation relative   to the use of Ezyfit under Matlab 8. |
| V2.41c | 2012/07/08 | **New function:**  - getlineinfo:   draws a line using the pointer, and displays the length and   angle of the segment in the figure.   **Bug fixes**  - showfit now recognizes   correctly if the Y-axis is in lin or log coordinates (thanks Jacopo).- showfit now conserves the 'hold on / off' state of the figure (thanks     Alec).- Equations containing a comma are now correctly recognized.       This allows to make use of functions like myfun(x,t) inside       a fit equation string (thanks Patrick).     - Installation of the Ezyfit menu now works properly under MacOS (thanks James Bagrow).   **Compatibility issue**:  - The standard location for the startup.m file has changed:   For Windows XP, it was in the matlabroot folder   (e.g. C:\Program Files\MATLAB\). It is now in the   userpath folder (e.g. C:\Users\Frederic\Documents\MATLAB).   Accordingly,   efmenu now creates or modifies the   startup.m file in the userpath folder. |
| V2.40 | 2010/07/20 | **New function**:  - remove\_efmenu\_fig:   This function removes the Ezyfit menu from figure files (.FIG).   This function allows one to use figure files generated from   a Matlab system with the Ezyfit toolbox on another Matlab system   without the Ezyfit toolbox. This fixes the issue of the Ezyfit   menu corrupting a figure file which could not be re-opened on a   Matlab system without Ezyfit installed. Many thanks to   Francis Burton and Nicholas Sinclair, who fixed   the issue in the Matlab Central Newsgroup.   **Minor changes**:  - The equation box is now displayed using the same font and   font size than the figure axes, with a truncation (see the Settings   page to change the tunctation length).   **Compatibility issue**:  - selecfit is removed from the toolbox. Use showfit   instead, with the "Data Brushing" tool (available for Matlab >= 7.6). See the release notes of V2.30   for details. - selectdata and delselectdata are also removed. |
| V2.30 | 2009/02/06 | **Major changes**:  - ezfit and showfit now support the new "Data Brushing" mode   of Matlab >= 7.6 (R2008a). When some data are "brushed" (i.e. when they have been   selected using the "Data brushing" tool), only those data are fitted by   ezfit and showfit. If you use an older version of Matlab   (version <=7.5, R2007b), this feature is not implemented, and all the   curve is selected. - Using showfit with "Brushed" data is equivalent to   selectfit. However, the combination showfit-"Data Brushing"   is more stable, since it relies on the Matlab's built-in Brush mode.   Since selectfit suffers from a number of bugs associated to the   lasso selection of data, selectfit is not going to be maintained   any more, and is going to be removed in future versions of EzyFit.   **New function**:  - ezfft: Easy to use FFT   (Power spectrum).   **New feature**:  - New Uninstallation page in the help browser. |
| V2.20 | 2008/03/08 | **Major changes**:  - The fit function has been renamed   ezfit, in order to avoid name conflicts with the "Curve Fitting   Toolbox" from MathWorks, Inc. The two toolboxes may now be used simultaneously. - "Magic initial guesses": When using predefined fits (e.g., showfit exp,   showfit power etc.), or the default fits in the EzyFit menu,   suitable default initial guesses are automatically chosen from the ranges   of data to be fitted. For instance, for a Gaussian fit, the initial guess   for the amplitude is taken as the max of the Y data, instead of 1. This   speeds up the convergence, and helps the solver when the true parameters are   too far from 1. These default values are ignored if other initial guesses   are provided by the user. - Weighted fits are now accepted for ezfit   (not yet for showfit and selectfit). See the Sample session   for an example. - The functions drawpolygon and selectdata have been   removed, and replaced with the more stable function selectdata   by John D'Errico (see Submission 13857   on the Matlab Central). As a result, a number of bugs of selectfit are   now fixed (see below). delseldata is now based on the new selectdata,   and keepseldata has been removed.   **New features**:  - New Sample session page in the help browser. - New predefined fits in the Ezyfit menu (e.g. 'a\*x^n+b', 'a\*(1-exp(-x/b))'...) - New item Refresh EzyFit menu in the menu, to update the user-defined fits   of a figure window when they have been changed from another figure window. - axis0 includes the origin in the axis. - axisl enlarges the axis of a log plot to include the nearest power of 10. - loglogpn and semilogypn: logarithmic plots for positive and negative Y data. - dfig creates a docked figure window. - New options figure and command for getslope.   **Bug fixes**:  - selectfit now works correctly on docked figure windows, or when legends are present.   However still buggy in some situations. - getslope now works correctly for Matlab >=7.4, and   works correctly when a legend is present. - showslope now issues an error message when used with docked windows (to be improved). Undock your window first. - showeqbox issued an error when 2nd parameter was not given. - Help from the 'Start' button now works correctly for Matlab >= 7.4.   **Minor changes**:  - The shortcut Ctrl-Z (Undo Fit) has been removed, because of a conflict with   Matlab's Undo operation.   **Compatibility issue**: Since Matlab 7.4 (R2007a), the behavior of the command doc has changed: the command doc ezyfit does not display the Ezyfit toolbox start page in the help browser any more. If you use Matlab >=7.4, type docezyfit to display the start page. Type docezyfit function\_name or showdemo function\_name to display the html documention of function\_name in the help browser. If you use Matlab 7.3 or before, doc and docezyfit will produce the same result. |
| V2.10 | 2006/10/30 | **Major upgrade**: Fit properties and display settings can now be passed to showfit and selectfit, such as fit colors, linewidth etc. For example: showfit('power','fitlinewidth',2,'fitcolor','green') The default properties are defined in fitparam. See the Settings page for details.  New options in fitparam: 'selectfitloopmode' and 'dispeqmode'; option 'eqdisplaymode' renamed 'eqreplacemode'.  By default, the polygon procedure in selectfit is done once; set the option selectfitloopmode to 'on' to repeat the procedure until an empty polygon is drawn (as in the previous versions).  fit now accepts row and column vectors for x and y.  Minor bug fixed: showfit and selectfit now accept a fit structure F even if the original curve has been deleted.  Minor bug fixed: when fitting histograms, the X points are now taken at the middle of the bins. |
| V2.04 | 2006/09/07 | New Frequently Asked Questions section.  Bug fixed: Selectfit now works when a legend box is present.  New options in fitparam: automakevarfit and whichpickdata.  fit, showfit and selectfit now also accept '!' and '$' instead of ';' to separate the list of options in the string FUN: this allows to use the syntax showfit power!log instead of showfit('power;log').  Minor bug fixed: showfit and selectfit now accept a fit structure F even if the original curve has been deleted.  New options in the item 'plotsample' of the Ezyfit menu. |
| V2.03 | 2006/04/12 | The 'EasyFit' toolbox has been renamed 'EzyFit', in order to avoid confusion with other products.  No change with EasyFit V2.02. |
| V2.02 | 2006/03/09 | Minor bugs fixed (the menus disappeared after a second call to plotsample; polynomial fits with unspecified orders was bugged).  'efmenu install' improved. |
| V2.01 | 2006/02/27 | **Major upgrade**: Left-hand side now accepted for the fitting equation: fit('e(k)=c/k^n') specifies that 'k' stands for the X-Data. If not specified, 'x' is taken by default.  The fitting equation is now case sensitive ('x' and 'X' are different).  New functions evalfit, makevarfit and showresidual.  Bug fixed: Matlab's standard legends are now correctly supported.  Capital greek letters, and greek letters as subscripts, are now correctly interpreted in the equation boxes. |
| V2.00 | 2006/02/10 | **Major change**: free parameter names may now be used with fit, showfit and selectfit, e.g., fit('a0+cste/x^n'), instead of fixed parameter names m1, m2, m3... The parameter names are automatically recognized.  New fit structure F.  New mat-files for predefined fits and user-defined fits.  New functions loadfit, editcoeff, showeqbox, dispeqfit.  Greek letters (lower case) are interpreted in the equation boxes (latex syntax).  Additional settings in fitparam.  Easyfit menu improved (Edit Fit Coefficients and Swap of the X- and Y- axis).  Better compatibility of the documentation with the Matlab help browser.  Function getfit retired (fit now does what getfit did). |
| V1.24 | 2006/01/30 | Bugs fixed in selectfit, drawpolygon and selectdata (better mouse button inquiries).  Bug fixed in efmenu (selectfit was wrong).  Showslope improved. |
| V1.23 | 2006/01/25 | User defined fits improved again (no need to type the fit number).  Check when fitting negative data in log mode. |
| V1.22 | 2006/01/12 | User defined fits in the Easyfit menu improved. |
| V1.21 | 2005/12/21 | Documentation improved. New 'Known bugs' section.  File 'Readme.txt' retired.  selectfit improved (message boxes). |
| V1.20 | 2005/12/15 | EasyFit menu and dialog boxes included.  Standard items for the toolbox in the Matlab 'Start' menu.  New about, checkupdate.  New home page (www.fast.u-psud.fr/ezyfit).  drawpolygon improved. |
| V1.14 | 2005/12/03 | drawpolygon improved: interactive properties of the window suspended during the drawing. |
| V1.13 | 2005/11/25 | Bug fixed in myginput for Matlab >=7.0.0. |
| V1.12 | 2005/11/03 | New parameter file 'paramfit.m'.  New drawpolygon, and selectdata improved.  getslope and showslope improved.  Now displays equations in Kaleidagraph or ABC mode. |
| V1.11 | 2005/10/31 | fit now computes the Pearson's correlation coefficient R.  n-th order polynomial fits added.  Initial guess may be defined within the fitting function string (as in Kaleidagraph).  New functions pickdata, undofit, rmfit, swx... |
| V1.10 | 2005/10/18 | Bug fixed for showfit.  New function selectfit, to select data by mouse in a polygon.  New function plotsample. |
| V1.05 | 2005/10/07 | New predefined fits (gaussian fits).  showfit now displays the equation in the plot.  New file 'readme.txt'. |
| v1.04 | 2005/07/27 |  |
| v1.00 | 2005/05/20 | First version. Originally called 'EasyFit toolbox'. |

  

|  |
| --- |
|  |

  
2005-2014 EzyFit Toolbox  
