# Supplementary material for: Inhibition Underlies Fast Undulatory Locomotion in Caenorhabditis elegans
Source: eNeuro. 2021 Mar 9;8(2):ENEURO.0241-20.2020. doi: 10.1523/ENEURO.0241-20.2020 (PMC7986531; doi:10.1523/ENEURO.0241-20.2020)
Supplement: Extended Data 1 — Code used in this study in three folders: (1) MATLAB program to plot curvature kymograms from hdf5 file generated by Tierpsy. (2) MATLAB program to analyze the change in fluorescence intensity of identifiable body-wall muscle cells or somata of motoneurons. (3) MATLAB code of computational models. Download Extended Data 1, ZIP file. [file enu-eN-NWR-0241-20-s13.zip › 2_CalciumImaging_Code/TrackAndMeasure_ImagingAnalyzer/ezyfit/html/ezyfit_settings.html]

EzyFit Settings


|  |
| --- |
| **EzyFit Settings** |

# EzyFit Settings

---

  

A number of fit properties and display settings (color, width,
equation box...) can be changed either:

- by specifying directly some 'PropertyName/PropertyValue' pairs,
  when calling showfit, e.g.

  ```
  showfit('a*x+b','fitcolor','red','fitlinewidth',1);
  ```
- by editing the default settings, which are coded in the
  M-file fitparam.m (click
  here to open this M-file and follow the
  instructions).

The actual settings structure can be displayed by simply typing fitparam.

This table lists the property names along with the type of values each accepts.
Curly braces { } enclose default values.

|  |  |  |
| --- | --- | --- |
| **Property Name** | **Property Values** | **Description** |
| fitcolor | [R G B] or *integer* or *string* | Color of the fit (type 'doc colorspec' for details):  - a [R G B] vector specifies a fixed color (eg, [0 0 0] is black).  - a string specifies one of eight predefined colors (full name: 'red', 'blue' etc, or single letters, 'r', 'b' etc).  - a numeric value specifies that the color of the fit has the same color as the data, but multiplied by the factor fitcolor (less than 1 for darker, larger than 1 for lighter).  Default = [1 0.5 0] (orange). |
| fitlinewidth | *integer* | Width of the fit line (type 'doc linespec' for details). Default = 2. |
| fitlinestyle | *string* | Style of the fit line (type 'doc linespec' for details). Default = '-'. |
| extrapol | fig | data | {none} | Extrapolation mode ('fig' = extrapolates the fit to the figure limits; 'data' = extrapolates the fit to the whole data limits, even if only a selection of the data is fitted (see selectfit); 'none' = no extrapolation) |
| npt | *integer* | Number of X points used to compute and display the fitted curve (typically 20 to 500, default=200) |
| dispeqmode | {on} | off | Display the equation in the command window. |
| dispeqboxmode | {on} | off | Display the equation box in the figure. |
| dispfitlegend | on | {off} | Display the legend (data and fits) in the figure |
| eqreplacemode | on | {off} | Equation replace mode ('off' = keep the parameter names in the equation; 'on' = replace each parameter by its numerical value in the equation). |
| corrcoefmode | {r} | r2 | none | Correlation coefficient mode: display R, R^2, or nothing. |
| linlogdisp | {on} | off | Lin/log mode display, tells whether Y or LOG(Y) is fitted. |
| editcoeffmode | on | {off} | Open the Array Editor with the fit coefficients after showfit. See editcoeff for details. |
| coeffarray | {row} | line | Array for the Fit Coefficients: 'row' or 'line' (this option is useful for copy-paste the coefficients in Excel) |
| automakevarfit | on | {off} | Call makevarfit after each fit. Setting to 'on' creates on the Matlab workspace the variables associated to the fit parameters (the variables will be overwritten if they already exist!). |
| maxlengtheq | *integer* | Maximum length of the equation string to be displayed in the equation box (longer strings are truncated). Set 'maxlength = inf' for no truncation. Default value = 35. |
| boxlocation | [x,y,w,h] | Location and size of the equation box (in normalized units). Default = [0.15 0.81 0.1 0.1]. |
| whichpickdata | {first} | last | Which data to fit by default when several curves are present in the figure and none is selected. |
| selectfitloopmode | on | {off} | Loop mode for selectfit ('on' = the polygon procedure is repeated until an empty polygon is drawn, and only the last trial is kept; 'off' = the polygon procedure is done once). Obsolete. |
| selectfitmsgbox | {on} | off | Message box display (instructions for drawing the polygon) in selectfit. Obsolete |
| markerselectpt | *string* | Markers used during the selection of the data (polygon) with selectfit (set to '' if you don't want the selected points to be displayed). Obsolete. |
| polynom\_coeffname | *string* | Name of the polynom coefficients. Using a '\_' (underscore) at the end of the coeff name allows for using the coefficient order as a subscript (as in latex syntax). Default: 'a\_'. |
| numberofdigit | *integer* | Number of digits for the coefficient values. Default = 5. |

  

|  |
| --- |
|  |

  
2005-2014 EzyFit Toolbox
