# Supplementary material for: Inhibition Underlies Fast Undulatory Locomotion in Caenorhabditis elegans
Source: eNeuro. 2021 Mar 9;8(2):ENEURO.0241-20.2020. doi: 10.1523/ENEURO.0241-20.2020 (PMC7986531; doi:10.1523/ENEURO.0241-20.2020)
Supplement: Extended Data 1 — Code used in this study in three folders: (1) MATLAB program to plot curvature kymograms from hdf5 file generated by Tierpsy. (2) MATLAB program to analyze the change in fluorescence intensity of identifiable body-wall muscle cells or somata of motoneurons. (3) MATLAB code of computational models. Download Extended Data 1, ZIP file. [file enu-eN-NWR-0241-20-s13.zip › 2_CalciumImaging_Code/TrackAndMeasure_ImagingAnalyzer/ezyfit/html/ezyfit_thanks.html]

PIVMat Acknowledgements


|  |
| --- |
| **PIVMat Acknowledgements** |

## Acknowledgements

---

Many thanks to Francis Burton and Nicholas Sinclair, who solved
the issue of the corrupted figure files containing the Ezyfit menu.

D. Doppler, C. Morize, J. Seiwert, D.J. Bilar, P. Erbach, D. Lueerssen, T. Mitchell, J. Wiley, S. Haddock, L. Kogan,
P. Kinnunen, T. Garcia-Nolen, A. de Zegher, J. Bagrow for suggestions and bug reports (although some are still on my to-do list!)

Suggestions and bug fixes from anonymous users of Matlab Central (Patrick, Rene)
are also acknowledged.

  

|  |
| --- |
|  |

  
2005-2014 EzyFit Toolbox  
