# Supplementary material for: Inhibition Underlies Fast Undulatory Locomotion in Caenorhabditis elegans
Source: eNeuro. 2021 Mar 9;8(2):ENEURO.0241-20.2020. doi: 10.1523/ENEURO.0241-20.2020 (PMC7986531; doi:10.1523/ENEURO.0241-20.2020)
Supplement: Extended Data 1 — Code used in this study in three folders: (1) MATLAB program to plot curvature kymograms from hdf5 file generated by Tierpsy. (2) MATLAB program to analyze the change in fluorescence intensity of identifiable body-wall muscle cells or somata of motoneurons. (3) MATLAB code of computational models. Download Extended Data 1, ZIP file. [file enu-eN-NWR-0241-20-s13.zip › 2_CalciumImaging_Code/TrackAndMeasure_ImagingAnalyzer/ezyfit/html/ezyfit_uninstall.html]

EzyFit Uninstallation


|  |
| --- |
| **EzyFit Uninstallation** |

# EzyFit Uninstallation

---

  

## Full uninstallation

In order to remove the Ezyfit toolbox from your Matlab installation,
follow the 3 steps:

1. Edit the 'startup file' by typing

> ```
> edit startup
> ```

and remove the following lines

> ```
> %   These lines have been added by 'efmenu install' (01-Apr-2008 23:10:11):
> efmenu;   % Includes the EzyFit menu for all new figure.
> fprintf(' To get started with the EzyFit toolbox, select EzyFit from the Help browser.\n\n');
> ```

2. From the menu 'File > Set Path', select the directory
'../ezyfit', and click on 'Remove' and 'Save'.

3. Restart Matab.

  

## Uninstallation of the Ezyfit menu only

If you wish to uninstall the Ezyfit menu from your figure windows
but you still want to use the command-line functions of the Ezyfit
toolbox:

Apply the steps 1 and 3 above.

  

## Uninstallation of the Ezyfit menu from saved figure files

If you have saved some figure files (.FIG) from a Matlab system
with the Ezyfit toolbox, the figure files include the Ezyfit menu.
As a consequence, those figure files cannot be opened with a Matlab
system without the Ezyfit toolbox.

It is possible to remove the Ezyfit menu from a figure file, using
the remove\_efmenu\_fig function.

  

|  |
| --- |
|  |

  
2005-2014 EzyFit Toolbox  
