# Supplementary material for: Inhibition Underlies Fast Undulatory Locomotion in Caenorhabditis elegans
Source: eNeuro. 2021 Mar 9;8(2):ENEURO.0241-20.2020. doi: 10.1523/ENEURO.0241-20.2020 (PMC7986531; doi:10.1523/ENEURO.0241-20.2020)
Supplement: Extended Data 1 — Code used in this study in three folders: (1) MATLAB program to plot curvature kymograms from hdf5 file generated by Tierpsy. (2) MATLAB program to analyze the change in fluorescence intensity of identifiable body-wall muscle cells or somata of motoneurons. (3) MATLAB code of computational models. Download Extended Data 1, ZIP file. [file enu-eN-NWR-0241-20-s13.zip › 2_CalciumImaging_Code/TrackAndMeasure_ImagingAnalyzer/ezyfit/html/fitparam.html]

fitparam (Ezyfit Toolbox)


|  |  |
| --- | --- |
| **EzyFit Function Reference** | **<< Prev** | **Next >>** |

fitparam  
Default settings for the EzyFit toolbox  
  
**Description**
```` ```
This M-File contains the default settings for the EzyFit Toolbox, 
such as the extrapolation mode, fit colors, equation box location etc. 
See the page 'Settings' in the help browser for the list of available 
settings. 
 
To change the default settings, edit this file (type: edit fitparam.m) 
and follow the instructions. You may also choose the item 
'Default Settings' in the EzyFit menu. 
 
FP = fitparam returns the structure FP containing the settings.
```

See Also

```
showfit, ezfit, showeqbox, dispeqfit, pickdata. 
 
Published output in the Help browser 
   showdemo fitparam
``` ````
  

|  |  |
| --- | --- |
| **Previous: ezfit** | **Next: getlineinfo** |

  
2005-2014 EzyFit Toolbox 2.42  
  
