# Supplementary material for: Inhibition Underlies Fast Undulatory Locomotion in Caenorhabditis elegans
Source: eNeuro. 2021 Mar 9;8(2):ENEURO.0241-20.2020. doi: 10.1523/ENEURO.0241-20.2020 (PMC7986531; doi:10.1523/ENEURO.0241-20.2020)
Supplement: Extended Data 1 — Code used in this study in three folders: (1) MATLAB program to plot curvature kymograms from hdf5 file generated by Tierpsy. (2) MATLAB program to analyze the change in fluorescence intensity of identifiable body-wall muscle cells or somata of motoneurons. (3) MATLAB code of computational models. Download Extended Data 1, ZIP file. [file enu-eN-NWR-0241-20-s13.zip › 2_CalciumImaging_Code/TrackAndMeasure_ImagingAnalyzer/ezyfit/html/getlineinfo.html]

getlineinfo (Ezyfit Toolbox)


|  |  |
| --- | --- |
| **EzyFit Function Reference** | **<< Prev** | **Next >>** |

getlineinfo  
Get information (length, angle) of a segment  
  
**Description**
```` ```
getlineinfo draws a line using the pointer, and displays the length and 
angle of the segment in the figure. The angle is in degrees by default, 
(zero angle for a horizontal line). 
 
[LEN, ANGLE, DX, DY] = getlineinfo returns the results. 
 
... = getlineinfo(OPT) specifies the angle property: OPT = 'rad', 'deg' 
or 'slope' (default is 'deg').  OPT = 'angle' shows only the angle. 
 
Note: The Image Processing Toolbox is required.
```

See Also

```
getslope, showslope. 
 
Published output in the Help browser 
   showdemo getlineinfo
``` ````
  

|  |  |
| --- | --- |
| **Previous: fitparam** | **Next: getslope** |

  
2005-2014 EzyFit Toolbox 2.42  
  
