# Supplementary material for: Inhibition Underlies Fast Undulatory Locomotion in Caenorhabditis elegans
Source: eNeuro. 2021 Mar 9;8(2):ENEURO.0241-20.2020. doi: 10.1523/ENEURO.0241-20.2020 (PMC7986531; doi:10.1523/ENEURO.0241-20.2020)
Supplement: Extended Data 1 — Code used in this study in three folders: (1) MATLAB program to plot curvature kymograms from hdf5 file generated by Tierpsy. (2) MATLAB program to analyze the change in fluorescence intensity of identifiable body-wall muscle cells or somata of motoneurons. (3) MATLAB code of computational models. Download Extended Data 1, ZIP file. [file enu-eN-NWR-0241-20-s13.zip › 2_CalciumImaging_Code/TrackAndMeasure_ImagingAnalyzer/ezyfit/html/getslope.html]

getslope (Ezyfit Toolbox)


|  |  |
| --- | --- |
| **EzyFit Function Reference** | **<< Prev** | **Next >>** |

getslope  
Slope of the current line.  
  
**Description**
```` ```
getslope displays the equation of the current line of the figure. Use 
the menu 'Insert > Line' to draw a line first. getslope allows for 
rough curve fitting "by eye". You may also use the shortcut Ctrl+G if 
the Ezyfit menu has been installed (see efmenu). 
 
Depending of the axis types, the equation of the line will be: 
     Y = N*X+A         for X linear and Y linear 
     Y = A*X^N         for X log and Y log 
     Y = A*EXP(N*X)    for X linear and Y log 
     Y = A+N*LOG(X)    for X log and Y linear (LOG = natural logarithm) 
 
getslope('Property1',...) specifies the display mode: 
     'figure'   output the result in the figure (by default) 
     'command'  output the result in the command window 
     'nodisplay'  no output 
     'slope'    displays only the slope N (by default). 
     'equation' displays the full equation (e.g., N*X+A) 
 
[N, A] = getslope(...) also returns the parameters N and A of the 
equation.
```

See Also

```
showslope, getlineinfo, plotsample, rmfit, efmenu. 
 
Published output in the Help browser 
   showdemo getslope
``` ````
  

|  |  |
| --- | --- |
| **Previous: getlineinfo** | **Next: gridc** |

  
2005-2014 EzyFit Toolbox 2.42  
  
