# Supplementary material for: Inhibition Underlies Fast Undulatory Locomotion in Caenorhabditis elegans
Source: eNeuro. 2021 Mar 9;8(2):ENEURO.0241-20.2020. doi: 10.1523/ENEURO.0241-20.2020 (PMC7986531; doi:10.1523/ENEURO.0241-20.2020)
Supplement: Extended Data 1 — Code used in this study in three folders: (1) MATLAB program to plot curvature kymograms from hdf5 file generated by Tierpsy. (2) MATLAB program to analyze the change in fluorescence intensity of identifiable body-wall muscle cells or somata of motoneurons. (3) MATLAB code of computational models. Download Extended Data 1, ZIP file. [file enu-eN-NWR-0241-20-s13.zip › 2_CalciumImaging_Code/TrackAndMeasure_ImagingAnalyzer/ezyfit/html/loadfit.html]

loadfit (Ezyfit Toolbox)


|  |  |
| --- | --- |
| **EzyFit Function Reference** | **<< Prev** | **Next >>** |

loadfit  
Load the predefined and the user-defined fitting functions.  
  
**Description**
```` ```
[DEFAULTFIT, USERFIT] = loadfit loads the predefined and the user- 
defined fitting functions. 
 
DEFAULTFIT = loadfit('default') loads only the predefined fits. 
USERFIT = loadfit('user') loads only the user-defined fits. 
 
DEFAULTFIT and USERFIT are structure arrays containing two fields, 
'name' and 'eq'. 
 
If the file(s) for the predefined and/or the user-defined fits do(es) 
not exist, loadfit creates it (them).
```

See Also

```
editfit, efmenu. 
 
Published output in the Help browser 
   showdemo loadfit
``` ````
  

|  |  |
| --- | --- |
| **Previous: liny** | **Next: loglogpn** |

  
2005-2014 EzyFit Toolbox 2.42  
  
