# Supplementary material for: Inhibition Underlies Fast Undulatory Locomotion in Caenorhabditis elegans
Source: eNeuro. 2021 Mar 9;8(2):ENEURO.0241-20.2020. doi: 10.1523/ENEURO.0241-20.2020 (PMC7986531; doi:10.1523/ENEURO.0241-20.2020)
Supplement: Extended Data 1 — Code used in this study in three folders: (1) MATLAB program to plot curvature kymograms from hdf5 file generated by Tierpsy. (2) MATLAB program to analyze the change in fluorescence intensity of identifiable body-wall muscle cells or somata of motoneurons. (3) MATLAB code of computational models. Download Extended Data 1, ZIP file. [file enu-eN-NWR-0241-20-s13.zip › 2_CalciumImaging_Code/TrackAndMeasure_ImagingAnalyzer/ezyfit/html/loglogpn.html]

loglogpn (Ezyfit Toolbox)


|  |  |
| --- | --- |
| **EzyFit Function Reference** | **<< Prev** | **Next >>** |

loglogpn  
Log-log scale plot for positive and negative data.  
  
**Description**
```` ```
loglogpn(...) is the same as LOGLOG(...), except that the Y data 
may have positive and negative values. This is useful to detect 
unexpected negative values in the Y data (causing repeated 'Negative 
data ignored' warnings), or to visualize the magnitude of an 
oscillating signal in log scale. 
 
loglogpn(X,Y) plots X versus Y in log-log scale, where Y may have 
positive and negative values. By default, negative values are ploted 
with dashed lines and positive values with full lines. If X is not 
specified, Y is plotted versus its index. 
 
loglogpn(X,Y,LineSpecPos,LineSpecNeg) specifies LineSpecPos and 
LineSpecNeg for the line types, marker symbols and colors for the 
positive and negative values of Y. For instance, 
loglogpn(X,Y,'ro-','b*') plots the positive Y with red line and 'o' 
markers and the negative Y with blue stars '*'. If only LineSpecPos 
is specified, the same is taken for LineSpecNeg, with a different 
LineStyle. 
 
loglogpn(X,Y,LineSpecPos,LineSpecNeg,'PropertyName',PropertyValue,...) 
sets property values for all lineseries graphics objects created by 
loglogpn. See the line reference page for more information. 
 
[HP, HN] = loglogpn(...) returns the handles to the two lineseries 
graphics objects.
```

Examples

```
   x = linspace(1,10,200); 
   y = sin(x*2)./x; 
   loglogpn(x,y,'r.','bo'); 
   axis([1 10 1e-2 1]); 
 
   loglogpn(x,y,'b-','r:','LineWidth',2); 
 
   [hp, hn] = loglogpn(x,y,'k'); 
   set(hp, 'LineWidth', 1); 
   set(hn, 'LineWidth', 2);
```

See Also

```
LOGLOG, SEMILOGY, semilogypn. 
 
Published output in the Help browser 
   showdemo loglogpn
``` ````
  

|  |  |
| --- | --- |
| **Previous: loadfit** | **Next: logx** |

  
2005-2014 EzyFit Toolbox 2.42  
  
