# Supplementary material for: Inhibition Underlies Fast Undulatory Locomotion in Caenorhabditis elegans
Source: eNeuro. 2021 Mar 9;8(2):ENEURO.0241-20.2020. doi: 10.1523/ENEURO.0241-20.2020 (PMC7986531; doi:10.1523/ENEURO.0241-20.2020)
Supplement: Extended Data 1 — Code used in this study in three folders: (1) MATLAB program to plot curvature kymograms from hdf5 file generated by Tierpsy. (2) MATLAB program to analyze the change in fluorescence intensity of identifiable body-wall muscle cells or somata of motoneurons. (3) MATLAB code of computational models. Download Extended Data 1, ZIP file. [file enu-eN-NWR-0241-20-s13.zip › 2_CalciumImaging_Code/TrackAndMeasure_ImagingAnalyzer/ezyfit/html/makevarfit.html]

makevarfit (Ezyfit Toolbox)


|  |  |
| --- | --- |
| **EzyFit Function Reference** | **<< Prev** | **Next >>** |

makevarfit  
Create variables from the parameters of a fit  
  
**Description**
```` ```
makevarfit(F) creates (in the Matlab workspace) the variables that 
contain the numerical values of the parameters from the fit F. 
 
If you want the variables to be automatically created in the Matlab 
workspace at each call of ezfit, showfit or selectfit, set the option 
'automakevarfit = on' in fitparam. 
 
If the input argument F is not specified, use the last fit.
```

Example

```
  Some sample data are fitted by a 2nd order polynom, and the 
  three variables 'a','b','c', which contain the numerical values 
  of the parameters, are created in the workspace: 
     plotsample('poly2'); 
     f = showfit('a*x^2+b*x+c'); 
     makevarfit(f); 
     whos
```

See Also

```
ezfit, showfit, editcoeff. 
 
Published output in the Help browser 
   showdemo makevarfit
``` ````
  

|  |  |
| --- | --- |
| **Previous: logy** | **Next: myginput** |

  
2005-2014 EzyFit Toolbox 2.42  
  
