# Supplementary material for: Inhibition Underlies Fast Undulatory Locomotion in Caenorhabditis elegans
Source: eNeuro. 2021 Mar 9;8(2):ENEURO.0241-20.2020. doi: 10.1523/ENEURO.0241-20.2020 (PMC7986531; doi:10.1523/ENEURO.0241-20.2020)
Supplement: Extended Data 1 — Code used in this study in three folders: (1) MATLAB program to plot curvature kymograms from hdf5 file generated by Tierpsy. (2) MATLAB program to analyze the change in fluorescence intensity of identifiable body-wall muscle cells or somata of motoneurons. (3) MATLAB code of computational models. Download Extended Data 1, ZIP file. [file enu-eN-NWR-0241-20-s13.zip › 2_CalciumImaging_Code/TrackAndMeasure_ImagingAnalyzer/ezyfit/html/myginput.html]

myginput (Ezyfit Toolbox)


|  |  |
| --- | --- |
| **EzyFit Function Reference** | **<< Prev** | **Next >>** |

myginput  
Graphical input from mouse with custum cursor pointer.  
  
**Description**
```` ```
[X,Y] = myginput(N) gets N points from the current axes and returns 
the X- and Y-coordinates in length N vectors X and Y. 
 
[X,Y] = myginput(N, POINTER) also specifies the cursor pointer, e.g. 
'crosshair', 'arrow', 'circle' etc. See "Specifying the Figure Pointer" 
in Matlab's documentation  to see the list of available pointers. 
 
myginput is strictly equivalent to Matlab's original GINPUT, except 
that a second argument specifies the cursor pointer instead of the 
default 'fullcrosshair' pointer.
```

Example

```
  plot(1:2,1:2,'s'); 
  hold on 
  [x,y] = myginput(1,'crosshair'); 
  plot(x,y,'o'); 
  hold off 
 
myginput is copied from Matlab's GINPUT rev. 5.32.4.4.
```

See Also

```
GINPUT. 
 
Published output in the Help browser 
   showdemo myginput
``` ````
  

|  |  |
| --- | --- |
| **Previous: makevarfit** | **Next: pickdata** |

  
2005-2014 EzyFit Toolbox 2.42  
  
