# Supplementary material for: Inhibition Underlies Fast Undulatory Locomotion in Caenorhabditis elegans
Source: eNeuro. 2021 Mar 9;8(2):ENEURO.0241-20.2020. doi: 10.1523/ENEURO.0241-20.2020 (PMC7986531; doi:10.1523/ENEURO.0241-20.2020)
Supplement: Extended Data 1 — Code used in this study in three folders: (1) MATLAB program to plot curvature kymograms from hdf5 file generated by Tierpsy. (2) MATLAB program to analyze the change in fluorescence intensity of identifiable body-wall muscle cells or somata of motoneurons. (3) MATLAB code of computational models. Download Extended Data 1, ZIP file. [file enu-eN-NWR-0241-20-s13.zip › 2_CalciumImaging_Code/TrackAndMeasure_ImagingAnalyzer/ezyfit/html/pickdata.html]

pickdata (Ezyfit Toolbox)


|  |  |
| --- | --- |
| **EzyFit Function Reference** | **<< Prev** | **Next >>** |

pickdata  
Picks data from the active curve.  
  
**Description**
```` ```
[X,Y] = pickdata returns the data (X,Y) from the active curve. If 
several curves are present and none is selected, the first one is 
picked (see fitparam to change this default setting). 
 
Specific feature for Matlab >= 7.6 : If some data are "brushed", return 
only those "brushed" data. 
 
[X,Y] = pickdata(H) returns the data (X,Y) of the curve specified by 
the handle H (eg, GCF, GCO etc). GCF, the current figure, is taken by 
default. 
 
For histograms, the X data are taken at the middle of the bins. 
 
[X,Y,H] = pickdata(...) also returns the handle to the current curve.
```

See Also

```
showfit, ezfit. 
 
Published output in the Help browser 
   showdemo pickdata
``` ````
  

|  |  |
| --- | --- |
| **Previous: myginput** | **Next: plotsample** |

  
2005-2014 EzyFit Toolbox 2.42  
  
