# Supplementary material for: Inhibition Underlies Fast Undulatory Locomotion in Caenorhabditis elegans
Source: eNeuro. 2021 Mar 9;8(2):ENEURO.0241-20.2020. doi: 10.1523/ENEURO.0241-20.2020 (PMC7986531; doi:10.1523/ENEURO.0241-20.2020)
Supplement: Extended Data 1 — Code used in this study in three folders: (1) MATLAB program to plot curvature kymograms from hdf5 file generated by Tierpsy. (2) MATLAB program to analyze the change in fluorescence intensity of identifiable body-wall muscle cells or somata of motoneurons. (3) MATLAB code of computational models. Download Extended Data 1, ZIP file. [file enu-eN-NWR-0241-20-s13.zip › 2_CalciumImaging_Code/TrackAndMeasure_ImagingAnalyzer/ezyfit/html/plotsample.html]

plotsample (Ezyfit Toolbox)


|  |  |
| --- | --- |
| **EzyFit Function Reference** | **<< Prev** | **Next >>** |

plotsample  
Display a sample plot.  
  
**Description**
```` ```
plotsample displays some noisy sample data. Try to fit the data with 
showfit or SELECTFIT, following the instructions given in the command 
window. The sample data is chosen randomly among the 10 predefined 
plots described below. 
 
plotsample is also available from the item 'Plot Sample' of the EzyFit 
menu (see efmenu). 
 
plotsample(OPT)  specifies the sample plot: 
  'power':   noisy power law 
  'linear':  noisy data to be fitted by a linear function 
  'osc':     oscillations 
  'damposc': damped oscillations 
  'cste':    noisy constant 
  'exp':     noisy exponential decay 
  'hist':    histogram of 1000 realizations of a random variable 
  'hist2':   histogram with two gaussian peaks. 
  'powco':   noisy power law with an exponential cut-off. 
  'poly2':   3 curves to be fitted by a 2nd order polynomial fit 
 
plotsample(N) specifies the number of the sample plot (1 to 10). 
 
plotsample(...,'fit') also displays the fit. 
plotsample(...,'nodisp') does not display the help text in the command 
window. 
 
[X,Y] = plotsample(...) also returns the sample data (this is 
equivalent to plotsample; [X,Y] = pickdata;).
```

Example

```
    plotsample power fit 
    (it shows a power law and fits it).
```

See Also

```
showfit, ezfit, pickdata, efmenu. 
 
Published output in the Help browser 
   showdemo plotsample
``` ````
  

|  |  |
| --- | --- |
| **Previous: pickdata** | **Next: remove\_efmenu\_fig** |

  
2005-2014 EzyFit Toolbox 2.42  
  
