# Supplementary material for: Inhibition Underlies Fast Undulatory Locomotion in Caenorhabditis elegans
Source: eNeuro. 2021 Mar 9;8(2):ENEURO.0241-20.2020. doi: 10.1523/ENEURO.0241-20.2020 (PMC7986531; doi:10.1523/ENEURO.0241-20.2020)
Supplement: Extended Data 1 — Code used in this study in three folders: (1) MATLAB program to plot curvature kymograms from hdf5 file generated by Tierpsy. (2) MATLAB program to analyze the change in fluorescence intensity of identifiable body-wall muscle cells or somata of motoneurons. (3) MATLAB code of computational models. Download Extended Data 1, ZIP file. [file enu-eN-NWR-0241-20-s13.zip › 2_CalciumImaging_Code/TrackAndMeasure_ImagingAnalyzer/ezyfit/html/remove_efmenu_fig.html]

remove\_efmenu\_fig (Ezyfit Toolbox)


|  |  |
| --- | --- |
| **EzyFit Function Reference** | **<< Prev** | **Next >>** |

remove\_efmenu\_fig  
Remove the Ezyfit menu from figure files (.FIG)  
  
**Description**
```` ```
remove_efmenu_fig(FILENAMEIN,FILENAMEOUT) removes the Ezyfit menu from 
the figure file FILENAMEIN, and saves the result under FILENAMEOUT. 
 
remove_efmenu_fig(FILENAMEIN) automatically generates the output 
filename, by adding the string '_new' to the input filename. 
 
This function fixes the issue of figure files including the Ezyfit menu 
opened in a Matlab system running without the Ezyfit toolbox. See 
efmenu for details.
```

Example

```
  plotsample;   % generate a random plot 
  saveas(gcf,'myfig.fig'); 
  remove_efmenu_fig('myfig.fig'); 
  % This generates a new file, named 'myfig_new.fig', without the menu 
 
Acknowledgments to Francis Burton and Nicholas Sinclair, who fixed 
the issue in the Matlab Central Newsgroup.
```

See Also

```
efmenu. 
 
Published output in the Help browser 
   showdemo remove_efmenu_fig
``` ````
  

|  |  |
| --- | --- |
| **Previous: plotsample** | **Next: rmfit** |

  
2005-2014 EzyFit Toolbox 2.42  
  
