# Supplementary material for: Inhibition Underlies Fast Undulatory Locomotion in Caenorhabditis elegans
Source: eNeuro. 2021 Mar 9;8(2):ENEURO.0241-20.2020. doi: 10.1523/ENEURO.0241-20.2020 (PMC7986531; doi:10.1523/ENEURO.0241-20.2020)
Supplement: Extended Data 1 — Code used in this study in three folders: (1) MATLAB program to plot curvature kymograms from hdf5 file generated by Tierpsy. (2) MATLAB program to analyze the change in fluorescence intensity of identifiable body-wall muscle cells or somata of motoneurons. (3) MATLAB code of computational models. Download Extended Data 1, ZIP file. [file enu-eN-NWR-0241-20-s13.zip › 2_CalciumImaging_Code/TrackAndMeasure_ImagingAnalyzer/ezyfit/html/rmfit.html]

rmfit (Ezyfit Toolbox)


|  |  |
| --- | --- |
| **EzyFit Function Reference** | **<< Prev** | **Next >>** |

rmfit  
Remove fits from the current figure  
  
**Description**
```` ```
rmfit removes all the fits (and their equation box) created by 
showfit from the current figure. 
 
rmfit('first') removes only the first fit. 
rmfit('last') removes only the last fit (equivalent to undofit). 
rmfit(N) removes only the Nth fit. 
rmfit('boxonly') removes only the equation boxes. 
rmfit('getslope') removes all the equations displayed by getslope and 
showslope. 
 
Note that rmfit does not remove the annotation objects (eg, lines).
```

See Also

```
showfit, undofit, getslope. 
 
Published output in the Help browser 
   showdemo rmfit
``` ````
  

|  |  |
| --- | --- |
| **Previous: remove\_efmenu\_fig** | **Next: semilogypn** |

  
2005-2014 EzyFit Toolbox 2.42  
  
