# Supplementary material for: Inhibition Underlies Fast Undulatory Locomotion in Caenorhabditis elegans
Source: eNeuro. 2021 Mar 9;8(2):ENEURO.0241-20.2020. doi: 10.1523/ENEURO.0241-20.2020 (PMC7986531; doi:10.1523/ENEURO.0241-20.2020)
Supplement: Extended Data 1 — Code used in this study in three folders: (1) MATLAB program to plot curvature kymograms from hdf5 file generated by Tierpsy. (2) MATLAB program to analyze the change in fluorescence intensity of identifiable body-wall muscle cells or somata of motoneurons. (3) MATLAB code of computational models. Download Extended Data 1, ZIP file. [file enu-eN-NWR-0241-20-s13.zip › 2_CalciumImaging_Code/TrackAndMeasure_ImagingAnalyzer/ezyfit/html/showeqbox.html]

showeqbox (Ezyfit Toolbox)


|  |  |
| --- | --- |
| **EzyFit Function Reference** | **<< Prev** | **Next >>** |

showeqbox  
Show the equation box of a fit.  
  
**Description**
```` ```
H = showeqbox(F) shows the equation box of the fit F, using the settings 
defined in fitparam. The fit structure F is obtained from ezfit. H is a 
handle to the equation box. 
 
Note that showeqbox interprets the greek symbols in the fitting equation 
(latex syntax). The '\' latex symbol must be omitted. 
 
showeqbox is automatically called from showfit or SELECTFIT when the 
option 'dispeqboxmode' is set to 'on' in the fitparam.m file.
```

Example

```
   plotsample('power') 
   f = ezfit('alpha/x^n'); 
   showeqbox(f);
```

See Also

```
fitparam, ezfit, showfit, dispeqfit. 
 
Published output in the Help browser 
   showdemo showeqbox
``` ````
  

|  |  |
| --- | --- |
| **Previous: semilogypn** | **Next: showeqbox\_new** |

  
2005-2014 EzyFit Toolbox 2.42  
  
