# Supplementary material for: Inhibition Underlies Fast Undulatory Locomotion in Caenorhabditis elegans
Source: eNeuro. 2021 Mar 9;8(2):ENEURO.0241-20.2020. doi: 10.1523/ENEURO.0241-20.2020 (PMC7986531; doi:10.1523/ENEURO.0241-20.2020)
Supplement: Extended Data 1 — Code used in this study in three folders: (1) MATLAB program to plot curvature kymograms from hdf5 file generated by Tierpsy. (2) MATLAB program to analyze the change in fluorescence intensity of identifiable body-wall muscle cells or somata of motoneurons. (3) MATLAB code of computational models. Download Extended Data 1, ZIP file. [file enu-eN-NWR-0241-20-s13.zip › 2_CalciumImaging_Code/TrackAndMeasure_ImagingAnalyzer/ezyfit/html/showfit.html]

showfit (Ezyfit Toolbox)


|  |  |
| --- | --- |
| **EzyFit Function Reference** | **<< Prev** | **Next >>** |

showfit  
Show the fit for the active curve.  
  
**Description**
```` ```
showfit displays the fit specified in a dialog box for the active 
curve. Use undofit or rmfit to remove the fit. The display settings are 
defined in the file fitparam. 
 
By default, the first curve in the active figure is used (see fitparam 
to change this default behavior). To fit another curve, select it 
before calling showfit. If some data are selected by the "Data 
Brushing" tool (only for Matlab >= 7.6), only those data are fitted. 
If you use Matlab <=7.5, use SELECTFIT to fit only a part of the data 
from the selected curve. 
 
showfit(FUN) specifies the fitting string FUN. FUN may be either a 
default fit (eg, 'exp', 'power'), a user-defined fit (see editfit), 
or directly a fit equation (eg, 'c+a*exp(-x/x0)'). See ezfit for the 
syntax of FUN. FUN may also be any valid interpolation method string 
(eg 'spline', 'cubic'... excepted 'linear'). See INTERP1 for the valid 
interpolation methods. 
 
showfit(F) displays the fit F defined from the fit structure returned 
by ezfit. See ezfit for the definition of F. 
 
showfit(..., 'PropertyName','PropertyValue',...) specifies the 
properties of the fit (e.g., fit colors, line width etc.). See the 
default values in the file fitparam. 
 
F = showfit(...) also returns the fit structure F. F has the same 
content as the fit structure returned by ezfit (see ezfit for details), 
and also contains a handle to the equation box and to the curve. 
 
Note that if the option 'lin' or 'log' is not specified in the string 
FUN, showfit checks the Y-scale to know if Y or LOG(Y) has to be 
fitted.
```

Examples

```
   type 'plotsample' and follow the instructions. 
 
   plotsample power 
   showfit('c*x^n; log'); 
 
   plotsample hist 
   f = ezfit('gauss'); 
   showfit(f,'fitcolor','red','fitlinewidth',2); 
 
   plotsample poly2 
   f = showfit('z(v) = poly3','dispfitlegend','on'); 
   editcoeff(f);
```

See Also

```
ezfit, undofit, rmfit, plotsample, pickdata, INTERP1. 
 
Published output in the Help browser 
   showdemo showfit
``` ````
  

|  |  |
| --- | --- |
| **Previous: showeqbox\_new** | **Next: showresidual** |

  
2005-2014 EzyFit Toolbox 2.42  
  
