# Supplementary material for: Inhibition Underlies Fast Undulatory Locomotion in Caenorhabditis elegans
Source: eNeuro. 2021 Mar 9;8(2):ENEURO.0241-20.2020. doi: 10.1523/ENEURO.0241-20.2020 (PMC7986531; doi:10.1523/ENEURO.0241-20.2020)
Supplement: Extended Data 1 — Code used in this study in three folders: (1) MATLAB program to plot curvature kymograms from hdf5 file generated by Tierpsy. (2) MATLAB program to analyze the change in fluorescence intensity of identifiable body-wall muscle cells or somata of motoneurons. (3) MATLAB code of computational models. Download Extended Data 1, ZIP file. [file enu-eN-NWR-0241-20-s13.zip › 2_CalciumImaging_Code/TrackAndMeasure_ImagingAnalyzer/ezyfit/html/showresidual.html]

showresidual (Ezyfit Toolbox)


|  |  |
| --- | --- |
| **EzyFit Function Reference** | **<< Prev** | **Next >>** |

showresidual  
Show the residuals of a fit  
  
**Description**
```` ```
showresidual(F) displays the residuals for the fit F in a new figure, 
ie: plots the difference between the data and the fit. 
 
showresidual is also available from the item 'Show Fit Residuals' in 
the EzyFit menu (see efmenu). 
 
If F is not specified, plots the residual for the last fit. 
 
YRES = showresidual(...) returns the residuals (YRES = YDATA - YFIT). 
 
showresidual(F, H) plots the residual in the figure H.
```

See Also

```
showfit, ezfit, efmenu. 
 
Published output in the Help browser 
   showdemo showresidual
``` ````
  

|  |  |
| --- | --- |
| **Previous: showfit** | **Next: showslope** |

  
2005-2014 EzyFit Toolbox 2.42  
  
