# Supplementary material for: Inhibition Underlies Fast Undulatory Locomotion in Caenorhabditis elegans
Source: eNeuro. 2021 Mar 9;8(2):ENEURO.0241-20.2020. doi: 10.1523/ENEURO.0241-20.2020 (PMC7986531; doi:10.1523/ENEURO.0241-20.2020)
Supplement: Extended Data 1 — Code used in this study in three folders: (1) MATLAB program to plot curvature kymograms from hdf5 file generated by Tierpsy. (2) MATLAB program to analyze the change in fluorescence intensity of identifiable body-wall muscle cells or somata of motoneurons. (3) MATLAB code of computational models. Download Extended Data 1, ZIP file. [file enu-eN-NWR-0241-20-s13.zip › 2_CalciumImaging_Code/TrackAndMeasure_ImagingAnalyzer/ezyfit/html/showslope.html]

showslope (Ezyfit Toolbox)


|  |  |
| --- | --- |
| **EzyFit Function Reference** | **<< Prev** | **Next >>** |

showslope  
Draw a line with fixed slope  
  
**Description**
```` ```
showslope(N) drags a line in the current figure with a fixed slope N 
(does not work with docked windows; undock your window first!) 
 
Depending of the axis types, the 'slope' N means: 
     Y = N*X+A         for X linear and Y linear 
     Y = A*X^N         for X log and Y log 
     Y = A*EXP(N*X)    for X linear and Y log 
     Y = A+N*LOG(X)    for X log and Y linear (LOG = natural logarithm) 
 
The value N is displayed close to the line. N may also be the string of 
any valid Matlab expression (eg, 'pi', '22/7', ...), which will be 
displayed close to the line. showslope(N,'nolabel') does not display 
the value N in the figure. 
 
H = showslope(..) also returns a handle to the line. 
 
By default, the line is an 'annotation object', ie: it is attached to 
the window and not to the figure axes. As a consequence, the line may 
be further moved, or may be used for getslope. However, resizing 
the window may in some case shift the line, and turning the axes from 
lin to log turns a power law to a log law... To avoid these problems, 
specify showslope(N,'fix'), which makes the line a real plot object 
attached to the figure axes.
```

See Also

```
getslope, getlineinfo, plotsample. 
 
Published output in the Help browser 
   showdemo showslope
``` ````
  

|  |  |
| --- | --- |
| **Previous: showresidual** | **Next: sw** |

  
2005-2014 EzyFit Toolbox 2.42  
  
