# Supplementary material for: Inhibition Underlies Fast Undulatory Locomotion in Caenorhabditis elegans
Source: eNeuro. 2021 Mar 9;8(2):ENEURO.0241-20.2020. doi: 10.1523/ENEURO.0241-20.2020 (PMC7986531; doi:10.1523/ENEURO.0241-20.2020)
Supplement: Extended Data Figure 2-3 — GABAergic mutant strains move at lower speed and frequency than wild-type 5 s poststimulus. Five seconds after a harsh touch to the head or tail, all GABAergic knock-out animals moved with lower translocation speed and undulation frequency than wild type. Calculated probabilities of the null hypotheses (p value) below 0.05 were considered significant and are in red. Download Figure 2-3, DOCX file. [file enu-eN-NWR-0241-20-s03.docx]

**Table 2-3.** GABAergic mutant strains move at lower speed and frequency than wild-type 5 seconds post-stimulus.

Five seconds after a harsh touch to the head or tail all GABAergic knockout animals moved with lower translocation speed and undulation frequency than wild type. Calculated probabilities for null hypotheses (p-value) below 0.05 were considered significant and are in red.

|  |  | **Translocation Speed (µm/s)*** | | | **Undulation Frequency (Hz)** | | |
| --- | --- | --- | --- | --- | --- | --- | --- |
|  |  | **Mean±SD** | **One-way ANOVA** | **p value (Tukey test)** | **Mean±SD** | **One-way ANOVA** | **p value (Tukey test)** |
| **Head Stimulation** | **Wild Type** | 345±51 | F(3,35)=67.78, p< 0.0001 | Comparison Reference | 0.45±0.06 | F(3,35)=13.70, p< 0.0001 | Comparison Reference |
|  | ***unc-25*** | 76±48 |  | < 0.0001 | 0.26±0.07 |  | 0.0004 |
|  | ***unc-46*** | 54±95 |  | < 0.0001 | 0.20±0.09 |  | <0.0001 |
|  | ***unc-47*** | 67±82 |  | < 0.0001 | 0.25±0.13 |  | 0.0002 |
| **Tail Stimulation** | **Wild Type** | 366±62 | F(3,35)=49.89, p< 0.0001 | Comparison Reference | 0.58±0.08 | F(3,35)=20.11, p< 0.0001 | Comparison Reference |
|  | ***unc-25*** | 131±101 |  | < 0.0001 | 0.30±0.11 |  | < 0.0001 |
|  | ***unc-46*** | 171±44 |  | < 0.0001 | 0.30±0.09 |  | < 0.0001 |
|  | ***unc-47*** | 143±32 |  | < 0.0001 | 0.33±0.06 |  | < 0.0001 |
| *Translocation speeds are the absolute values regardless of moving directions. | | | | | | | |
